# A review of PI3K/AKT/mTOR inhibitors from traditional Chinese medicine: potential and perspective for activated phosphoinositide 3-kinase δ syndrome

**DOI:** 10.1186/s13023-026-04437-9

**Published:** 2026-06-16

**Authors:** Yixuan Xie, Linghui Nie, Litao Bai, Xiaodong Zhao

**Affiliations:** 1 Beijing University of Chinese Medicine, Beijing, China; 2 Children’s Behavioral Development Rehabilitation Center, Guangdong Second Provincial General Hospital, Guangzhou, Guangdong China; 3 Department of Integrated Chinese and Western Medicine, The Second Affiliated Hospital of Chongqing Medical University, Chongqing, China; 4 Chongqing Rong Guan technology co., LTD, Chongqing, China; 5 Department of Rheumatology and Immunology, Children’s Hospital of Chongqing Medical University, National Clinical Research Center for Child Health and Disorders, Ministry of Education Key Laboratory of Child Development and Disorders, Chongqing, China; 6 Chongqing Key Laboratory of Child Rare Diseases in Infection and Immunity, Chongqing, China

**Keywords:** Activated phosphoinositide 3-kinase δ syndrome, PI3K/AKT/mTOR pathway, Traditional chinese medicine, Immunomodulation

## Abstract

Activated phosphoinositide 3-kinase δ syndrome (APDS) is a primary immunodeficiency caused by hyperactivation of the PI3K/AKT/mTOR pathway, resulting in severe lymphoproliferation, recurrent infections, autoimmunity, and malignancy. However, current therapies, including immunosuppressants and hematopoietic stem cell transplantation, are constrained by limited efficacy, high costs, donor scarcity, or adverse effects such as metabolic complications from chronic rapamycin use. In this review, we systematically synthesize preclinical evidence on traditional Chinese medicine (TCM) agents that target the PI3K/AKT/mTOR pathway. Our goal is to establish a hypothesis-driven framework for APDS drug discovery. Through a comprehensive PubMed, CNKI, and Wanfang databases up to October 30, 2025, we identified 332 eligible studies, compiling a repository of 5 herbs, 99 extracts, and 56 formulas. These agents, rich in flavonoids, terpenoids, alkaloids, and polysaccharides, exhibit multifaceted pharmacological activities such as antitumor, anti-inflammatory, and immunomodulatory effects. Notably, 15 candidates show promise in ameliorating lymphoproliferation, infection, and gastrointestinal dysfunction in disease models with pathophysiological overlap with APDS. However, we emphasize that all current evidence is derived from non-APDS experimental systems; direct validation in patient-derived immune cells or APDS-mutant models remains absent. This review therefore provides a prioritized, evidence-based repository of testable hypotheses and a translational roadmap, advocating for rigorous functional validation, isoform-specific mechanistic dissection, and integration with conventional therapies to bridge the gap between traditional medicine and precision immunotherapy for APDS.

**Supplementary Information:**

The online version contains supplementary material available at https://doi.org/10.1186/s13023-026-04437-9.

## Background

Activated phosphoinositide 3-kinase δ syndrome (APDS) is a primary immunodeficiency disease caused by gain-of-function mutations in PIK3CD or PIK3R1. These mutations lead to constitutive activation of the PI3Kδ subunit, resulting in hyperactivation of the PI3K/AKT/mTOR signaling pathway—a key driver of disease pathogenesis [1–5]. Clinically, APDS presents with a spectrum of manifestations including recurrent respiratory infections, herpesviruses, lymphoproliferation, autoimmune cytopenias and enteropathy. Common features encompass chronic cough, diarrhea, lymphadenopathy, splenomegaly, and an elevated risk of lymphoma and bronchiectasis [1, 5–7]. These multisystem complications profoundly impact quality of life and long-term outcomes.

Current treatments for APDS include anti-infective prophylaxis, immunoglobulin replacement therapy (IRT), immunosuppressive agents such as glucocorticoids and rapamycin, and hematopoietic stem cell transplantation (HSCT) in severe cases. While rapamycin can mitigate lymphoproliferation and IRT reduces infection-related morbidity, limitations remain: rapamycin shows minimal efficacy against enteropathy, IRT is costly and not universally accessible, and HSCT is constrained by donor availability and procedural risks [8–10]. Thus, there is a clear need for more effective, safe, and broadly available treatment strategies.

TCM has been used in the management of various immune-related and chronic inflammatory conditions, including recurrent infections and gastrointestinal symptoms [11–16]. Notably, a growing body of preclinical evidence indicates that specific TCM-derived compounds and formulations can inhibit PI3K/AKT/mTOR signaling in models of cancer and inflammatory disease [17–19].The concordance between the known pharmacological effects of TCM (e.g., anti-inflammatory, immunomodulatory, and antitumor) and the key pathophysiological processes of APDS warrants the hypothesis that TCM may have therapeutic relevance.

However, direct evidence supporting TCM use in APDS remains extremely limited. This review aims to systematically collect and analyze TCM formulations, including herbs, extracts, and formulas, that inhibit the PI3K/AKT/mTOR pathway. By synthesizing their documented therapeutic effects in conditions that share symptomatologic and mechanistic features with APDS, this work aims to catalog candidate TCM compounds and establish a knowledge foundation for future mechanistic and translational research in APDS.

## Method

### Literature retrieval

**Fig. 1:**
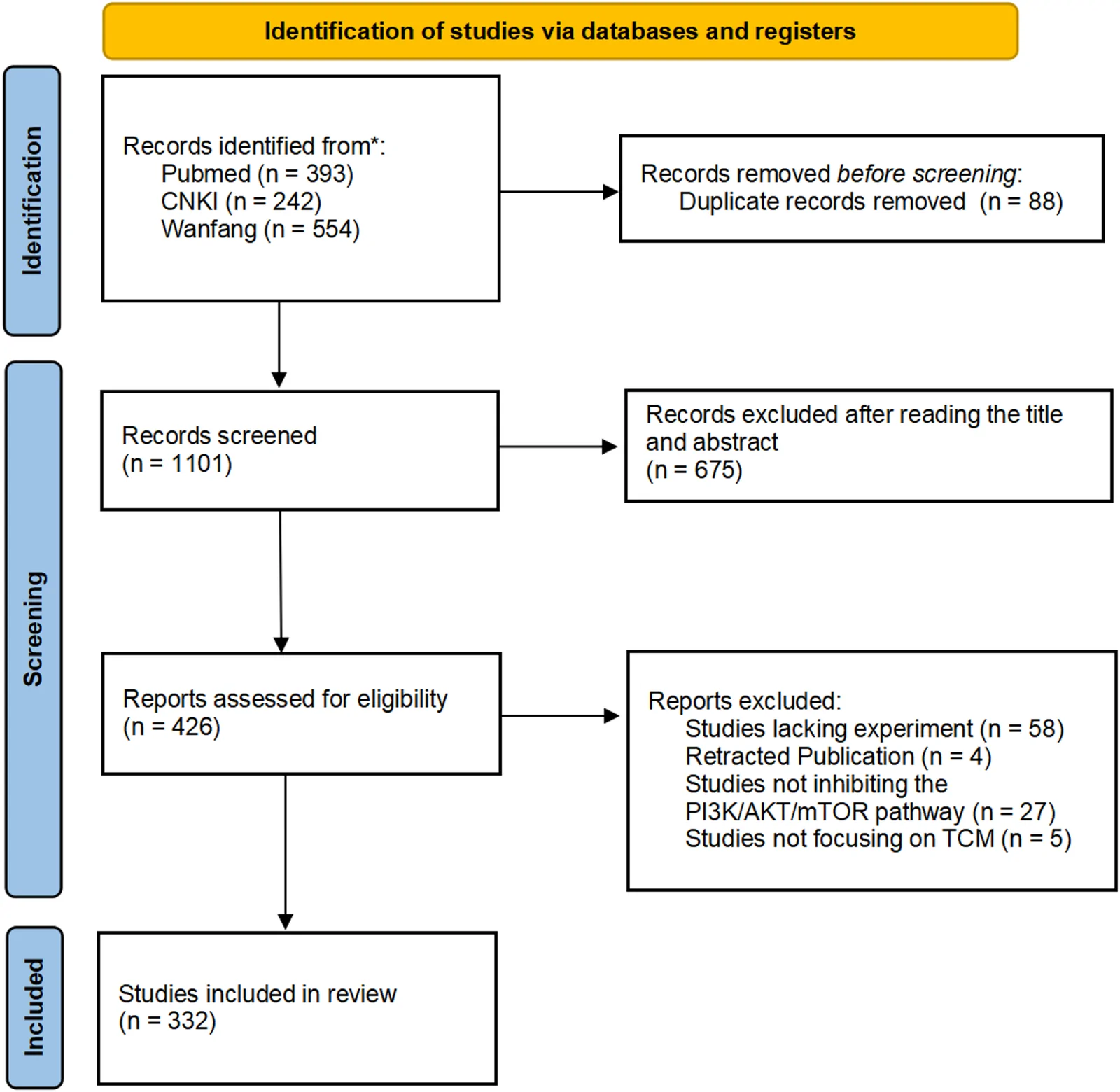
Retrieval process

We searched PubMed, CNKI, and Wanfang databases from their inception to October 30, 2025, to identify eligible studies. The search strategies and results were in Supplementary Table 1. Our systematic search yielded 1189 citations, which, after deduplication, reduced to 1101. Following title and abstract screening, 675 citations were excluded for not meeting the inclusion criteria. Of the 426 full-text articles reviewed, 94 were further excluded due to various reasons, leaving 332 studies for the review. This process is depicted in Fig. 1.

### Inclusion and exclusion criteria

To ensure the relevance and quality of the included articles, we established the following criteria.

#### Inclusion criteria

Studies focusing on herbs, extracts, or formulated prescriptions.

Studies investigating the inhibition of the PI3K/AKT/mTOR pathway or related targets.

Studies providing evidence from experimental validation or clinical trials.

#### Exclusion criteria

Studies not focusing on herbs, extracts, or formulated prescriptions.

Studies not targeting the PI3K/AKT/mTOR pathway or mechanisms related to APDS.

Studies lacking experimental validation or clinical trials to support the therapeutic effects of TCM interventions, including literature reviews or network pharmacology studies without empirical evidence.

Studies not published in English or Chinese.

### Key information extraction and analysis

Two independent reviewers screened the titles and abstracts of the identified articles to determine their eligibility against the inclusion and exclusion criteria. For those articles that met the criteria, full-text versions were retrieved and further assessed for relevance and key information extraction. Key information was extracted using a standardized form, including the types of herbs, extracts derived from TCM, and formulas, along with their respective sources, extraction methods, experimental dosages, molecular targets within the PI3K/AKT/mTOR pathway, and associated functional effects (Supplementary Tables 2–11).

## Systematic identification and prioritization of PI3K/AKT/mTOR targeting TCM candidates

We categorize different traditional Chinese medicine (TCM) inhibitors into three groups: herbs, Chinese medicine extracts, and Chinese medicine formulas, to facilitate a more systematic study and evaluation of their effects on inhibiting the PI3K/AKT/mTOR pathway and its targets.

### Herbs

Our systematic search identified five single herbs reported to inhibit the PI3K/AKT/mTOR signaling pathway (Supplementary Table 2). These herbs suppress key components of the PI3K/AKT/mTOR pathway, including p-PI3K, p-AKT, and p-mTOR. Their effects have been observed in cancer and inflammation models. The reported pharmacological activities include anti-proliferation, pro-apoptosis, anti-inflammation, and anti-fibrosis [20–24]. Notably, studies on Aconitum pendulum Busch in an autoimmune arthritis model suggest a pathological relevance to APDS [22]. However, none of these herbs have been directly validated in APDS-specific immunological models, highlighting a critical gap for future research.

### Chinese medicine extract

Our systematic analysis identified 99 botanical extracts with inhibitory activity against the PI3K/AKT/mTOR pathway, which were systematically categorized by chemical structure into 31 flavonoids, 21 terpenoids, 4 steroids, 8 alkaloids, 12 phenols, 11 quinones, 3 lignans, and 9 other or unknown extracts (Supplementary Tables 3–10). These compounds are derived from medicinal plants, as well as common fruits and vegetables. In various preclinical models, they exhibit a broad spectrum of pharmacological activities, including antitumor, anti-inflammatory, anti-proliferative, pro-apoptotic, autophagy-inducing, and immunomodulatory effects. Their mechanisms involve the downregulation of key proteins in the PI3K/AKT/mTOR pathway (e.g., p-PI3K, p-AKT, p-mTOR) and modulation of downstream targets such as Bcl-2, Bax, and LC3 [25–122]. Experimental doses ranged from nanomolar to micromolar concentrations, and most compounds were commercially sourced with a high degree of standardization. Notably, certain active constituents, including baicalein [26, 123, 156, 159, 176] and quercetin [27, 46, 91, 123, 148, 156, 158, 175–176, 178], have been repeatedly reported across independent studies as potent and reliable inhibitors of this pathway.

### Chinese medicine formulas

This study systematically compiled 56 Chinese medicine formulas with demonstrated inhibitory effects on the PI3K/AKT/mTOR signaling pathway (Supplementary Table 11). These formulas exert their effects through synergistic actions on multiple targets, showing capabilities in suppressing proliferation, promoting apoptosis, inducing autophagy, and reducing inflammation and invasion in various preclinical models [123–178]. Commonly used herbs include Scutellaria barbata, Astragalus membranaceus, Glycyrrhiza uralensis, and Curcuma zedoaria, with shared bioactive ingredients such as quercetin, baicalein, calycosin, and formononetin frequently appearing across formulas.

In summary, cross-formula analysis shows that many formulas inhibit the PI3K/AKT/mTOR pathway through a recurring set of key bioactive constituents. This convergence underscores the reliability of these compounds as pathway inhibitors. For instance, the widespread use of Astragalus membranaceus introduces active compounds like calycosin and astragalosides into numerous formulas such as Jianpi Yangzheng and Weipixiao decoctions [127–128]. Similarly, baicalein from Scutellaria baicalensis is a common component in formulas like Qingyihuaji and Shaoyao decoctions, while ginseng saponins and rhubarb-derived quinones (e.g., emodin, rhein) also recurrently contribute to pathway suppression [139, 142, 148, 149, 151, 154, 156, 161, 169, 172–173]. The recurrence of these bioactives across diverse contexts underscores their reliability as PI3K/AKT/mTOR pathway inhibitors, prioritizing them for future research.

### Integrated screening and prioritization of promising TCM candidates for APDS

#### Prioritization of promising candidates

To transition from a systematic cataloguing of TCM agents to a critical identification of the most promising candidates for APDS, we established and applied a predefined evidence assessment framework (Supplementary Method). This framework evaluates five core dimensions—model correlation, mechanistic depth, data robustness, safety profile, and standardization level—each rated as High (H), Medium (M), or Low (L) according to detailed criteria in Supplementary Table 12.

Briefly, model correlation assesses the relevance of the experimental model to APDS pathophysiology, ranging from APDS patient-derived cells and symptom-relevant models to generic cancer cell lines; mechanistic depth evaluates the extent of target validation, from direct binding assays and genetic manipulation to Western blotting or network pharmacology alone; data robustness reflects experimental rigor, including dose–response studies, positive controls, and reproducibility across models; safety profile considers toxicity data and therapeutic window, from in vivo pharmacokinetics to unassessed safety; and standardization level addresses the quality control of TCM preparations, from pure compounds with defined purity to uncharacterized crude extracts. These dimensions are grounded in established principles of preclinical drug evaluation [179–181]. To derive an overall intensity rating, we applied the rules detailed in Supplementary Method, with model correlation and mechanistic depth as core dimensions and safety as a critical limiting factor.

Applying these screening criteria, we identified a high-priority list of candidates (Table 1). Dihydromyricetin emerges as a prominent candidate for APDS-associated enteropathy. It demonstrates potent anti-inflammatory and autophagy-inducing effects in models of diarrhea and inflammation, and shows a close association with the PI3K/AKT/mTOR pathway [55]. Similarly, the flavonoid ampelopsin alleviates infection-induced inflammation through PI3K/AKT inhibition. It also ameliorates autoimmune predisposition by suppressing NF-κB [48]. Among terpenoids, celastrol alleviated rheumatoid arthritis by inhibiting PI3K/AKT/mTOR and inducing autophagy. Meanwhile, andrographolide attenuates sepsis-induced lung injury through RAGE receptor binding, which suppresses PI3K/AKT/mTOR signaling and NLRP3 inflammasome activation [61, 67]. The alkaloid extract of Toddalia asiatica shows anti-inflammatory effects in cellular models, consistent with APDS-related immune hyperactivation [87]. The phenolic compound epigallocatechin-3-gallate suppresses inflammation and cell migration in models of rheumatoid arthritis [95]. Furthermore, the quinone rhein demonstrates broad anti-inflammatory activity [111], and Prunella vulgaris extract shows efficacy in lymphoma models—a complication highly relevant to APDS [122].

Among formulas, several demonstrate targeted potential against specific APDS clinical phenotypes. Compound 511 stands out due to direct evidence showing it ameliorates immune deficiency and infection susceptibility. It restores the Th1/Th2 and Th17/Treg balance via the PI3K/AKT/mTOR pathway and reduces pneumonia severity and mortality [148]. For gastrointestinal complications such as enteropathy, Shaoyao Decoction, Chang-An-Jun-Tai Decoction, and Xiang Lian Pill have demonstrated efficacy in models of ulcerative colitis and irritable bowel syndrome, alleviating intestinal inflammation and enhancing barrier integrity, which effects with clear translational relevance to APDS [156, 177–178]. For respiratory manifestations, Zi-Su-Zi decoction and Pingchuan Granules confer protection in models of cough variant asthma and airway inflammation. They achieve this through anti-inflammatory mechanisms [143, 166]. Furthermore, Compound Kushen Injection exhibits potent anti-tumor activity in lymphoma models, underscoring its potential utility in managing APDS-associated lymphoproliferation [165].

Collectively, these candidates constitute a promising therapeutic arsenal characterized by precise target engagement and robust preclinical support, offering a multifaceted strategy to modulate innate immune dysregulation in APDS and warranting further validation in disease-specific experimental systems.

**Table 1:** Top-priority TCM-derived candidates for future APDS research

Category	Name	Model correlation	Mechanism depth	Data Robustness	Safety	Standardization level	Overall intensity	Mechanism	Function	Reference
Flavonoids	Dihydromyricetin (DHM)	M	H	H	M	H	H	p-PI3K↓, p-AKT↓, p-mTOR↓, LC3A/B↑	Inflammation ↓, Fibrosis ↓, Autophagy ↑	[55]
Flavonoids	Ampelopsin (Amp)	M	H	M	M	H	H	p-AKT↓, NF-κB↓	Inflammation ↓	[48]
Terpenoids	Celastrol (Cel)	M	H	M	M	H	H	p-PI3K↓, p-AKT↓, p-mTOR↓, LC3-II/LC3-I↑, Beclin-1↑	Proliferation ↓, autophagy ↑, inflammation ↓	[61]
Terpenoids	Andrographolide (And)	M	H	H	M	H	H	p-PI3K↓, AKT↓, p-mTOR↓	Autophagy ↑, inflammation ↓	[67]
Alkaloids	Toddalia asiatica alcohol extract (TAAE)	M	H	M	M	M	H	p-PI3K↓, p-AKT↓, p-mTOR↓, LC3-II/I↑, Beclin1↑, Atg5↑, p62↓, TNF-α↓, IL−6↓, COX−2↓	Autophagy↑, inflammation↓, macrophage activation↓, immune regulation	[87]
Phenols	Epigallocatechin-3-gallate (EGCG)	M	H	M	M	H	H	p-PI3K↓, p-AKT↓	Proliferation ↓, invasion ↓, migration ↓, apoptosis ↑, inflammation ↓	[95]
Quinones	Rhein	M	M	H	M	H	H	p-PI3K↓, p-AKT↓, p-mTOR↓, p-p70S6K↓	Inflammation ↓	[111]
Other classes of drugs or unknown	Prunella vulgaris extract (PVE)	M	M	H	M	M	H	p-AKT↓, p-mTOR↓	Proliferation ↓, apoptosis ↑	[122]
Formulas	Zi-Su-Zi decoction (ZSD)	M	M	M	M	M	H	p-PI3K↓, p-AKT1↓, p-mTOR↓	Inflammation ↓, allergy ↓	[143]
Formulas	Compound 511 (C511)	M	M	H	M	H	H	p-PI3K↓, p-AKT↓, p-mTOR↓	Inflammation ↓, infection ↓, immune regulation	[148]
Formulas	Shaoyao Decoction (SYD)	M	M	M	M	H	H	p-PI3K ↓, p-AKT ↓, p-mTOR ↓, AKT1 ↓	Inflammation ↓, apoptosis ↑	[156]
Formulas	Compound Kushen Injection (CKI)	M	M	H	M	M	H	PI3K↓, p-AKT↓, AKT↓, mTOR↓, p-mTOR↓	Proliferation↓, apoptosis↑, immunomodulation	[165]
Formulas	Pingchuan Granules (PCG)	M	H	M	M	M	H	p-AKT↓, p-mTOR↓	Autophagy↓, inflammatory↓, asthmatic↓	[166]
Formulas	Chang-An-Jun-Tai Decoction (CAJT)	M	H	M	M	M	H	p-PI3K↓, p-AKT↓, p-mTOR↓, PI3K mRNA↓, AKT mRNA↓, mTOR mRNA↓	Antidiarrheal↑, inflammation↓	[177]
Formulas	Xiang Lian Pill (XLP)	M	M	H	M	M	H	p-PI3K↓, p-AKT↓, p-mTOR↓, NF-κB p65↓, HIF-1α↓, LC3↑, Beclin-1↑	Inflammation↓, autophagy↑	[178]

(H: High, M: Medium, L: Low)

#### Comparative analysis of mechanisms of action

**Fig. 2:**
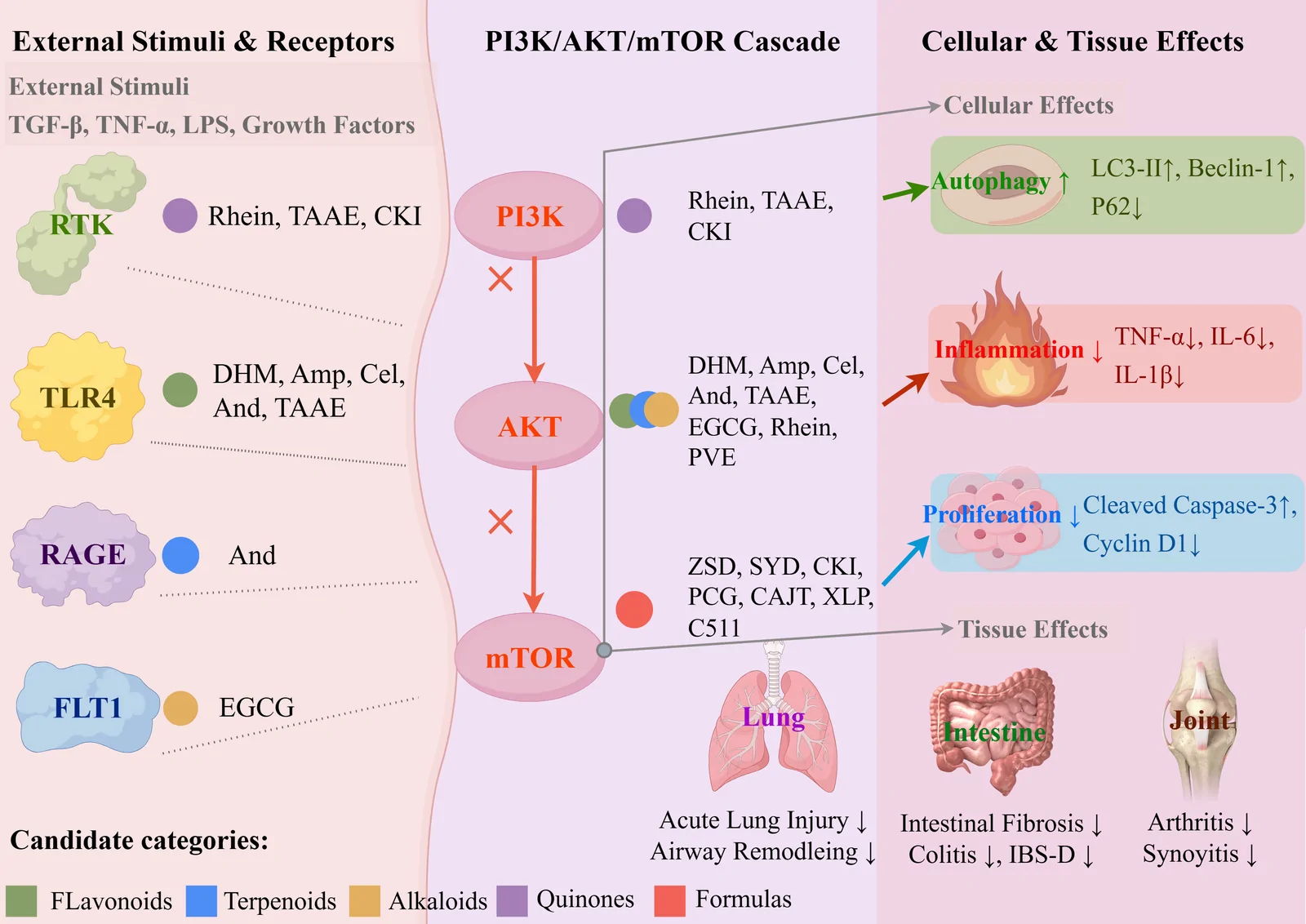
Proposed mechanisms and putative action sites of prioritized TCM candidates on the PI3K/AKT/mTOR pathway for APDS therapy. (DHM: Dihydromyricetin, Amp: Ampelopsin, Cel: Celastrol, And: Andrographolide, TAAE: Toddalia asiatica alcohol extract, EGCG: Epigallocatechin-3-gallate, PVE: Prunella vulgaris extract, ZSD: Zi-Su-Zi decoction, C511: Compound 511, SYD: Shaoyao Decoction, CKI: Compound Kushen Injection, PCG: Pingchuan Granules, CAJT: Chang-An-Jun-Tai Decoction, XLP: Xiang Lian Pill, × and ↓: Inhibition, ↑: Activation)

The top-priority TCM-derived candidates all target the PI3K/AKT/mTOR pathway. However, their specific sites of action and resulting pharmacological profiles vary considerably (Fig. 2). The flavonoid Dihydromyricetin acts at a mid-stream level by directly suppressing AKT and mTOR phosphorylation. This relieves mTOR-mediated inhibition of autophagy, leading to anti-fibrotic and anti-inflammatory effects. These properties make it particularly relevant for conditions such as enteropathy-associated tissue remodeling [55]. Ampelopsin, another flavonoid, acts further upstream by scavenging reactive oxygen species (ROS). This inhibits Akt phosphorylation, which in turn suppresses IKK/IκB phosphorylation and NF-κB nuclear translocation. The result is potent anti-inflammatory and antioxidant activities suited for infection-driven hyperinflammation [48]. Among terpenoids, Celastrol directly suppresses the PI3K/AKT/mTOR axis by inhibiting key phosphorylation events and potently inducing autophagic flux. Molecular evidence includes elevated LC3-II and Beclin-1 expression, along with reduced p62 accumulation. These changes collectively attenuate synovial proliferation and inflammatory responses in rheumatoid arthritis models [61]. Andrographolide, in contrast, initiates its action at the receptor level by binding RAGE. This leads to downstream suppression of PI3K/AKT/mTOR signaling and subsequent NLRP3 inflammasome inactivation, a mechanism beneficial in sepsis-induced lung injury [67]. The alkaloid-rich Toddalia asiatica alcohol extract (TAAE) and the quinone Rhein both primarily inhibit upstream PI3K phosphorylation. However, Rhein additionally demonstrates a unique capacity to remodel the gut microbiota, adding ecological regulation to its anti-inflammatory profile [87, 111]. Similarly, Prunella vulgaris extract and Compound Kushen Injection potently suppress PI3K/AKT/mTOR signaling. They also promote caspase-dependent apoptosis and impair tumor energy metabolism, indicating high relevance for lymphoproliferative complications [122, 165]. The phenolic compound Epigallocatechin-3-gallate (EGCG) acts by inhibiting the upstream receptor FLT1. This reduces PI3K/AKT activation, thereby suppressing synovial cell proliferation, migration, and inflammation [95]. Complex formulations such as Zi-Su-Zi decoction [143], Compound 511 [148], Shaoyao Decoction [156], Pingchuan Granules [166], Chang-An-Jun-Tai Decoction [177], and Xiang Lian Pill [178] exemplify a systems-level approach. Through synergistic multi-component actions, they concurrently modulate multiple intersecting pathways—including PI3K/AKT/mTOR, JAK2/STAT3, NF-κB, and HIF-1α. This results in broad immunomodulatory, anti-inflammatory, autophagy-modulating, and barrier-restoring effects. This mechanistic stratification has important implications. It suggests that targeting the diverse clinical manifestations of APDS—such as lymphoma versus enteropathy—may require selectively leveraging candidates with distinct hierarchical actions. Alternatively, it may call for rationally designed combination therapies tailored to specific pathological processes.

#### Critical appraisal of current evidence and its limitations

Our integration of the available evidence reveals several pervasive limitations that must be addressed to advance the field. First, regarding model relevance, the current body of evidence is predominantly derived from non-APDS models, such as generic cancer cell lines or broad inflammatory models. Consequently, the extrapolation of these findings to APDS-specific immune dysregulation, particularly to aberrant T and B cell functions, lacks experimental validation. Second, concerning mechanistic depth, the majority of studies remain observational, primarily documenting changes in pathway phosphorylation via Western blotting. Furthermore, there is a notable scarcity of studies investigating the selective inhibition of the PI3Kδ isoform, which is the core pathogenic subunit in APDS, or providing conclusive evidence derived from direct target engagement assays, such as binding experiments. Third, standardization and reproducibility pose significant challenges. The preparation methods and compositional profiles of TCM extracts and formulas vary substantially across different studies. This heterogeneity complicates direct comparison, hinders the accurate attribution of pharmacological effects, and ultimately impedes clinical translation.

## Discussion

The PI3K/AKT/mTOR signaling pathway has been increasingly implicated as a central pathogenic mechanism in APDS, forming a rational basis for therapeutic intervention. However, the rarity of this immunodeficiency has resulted in a notable scarcity of research exploring targeted therapies beyond conventional approaches. In response, this review has systematically consolidated the existing literature and has established, for the first time, a comprehensive database of Chinese medicinal herbs, extracts, and formulated products with confirmed activity in suppressing this pathway (Supplementary Tables 2–11). By applying a predefined prioritization framework, we have distilled a shortlist of high-priority candidates whose multifaceted pharmacological profiles exhibit compelling congruence with the complex pathophysiology of APDS (Table 1). These profiles encompass anti-inflammatory, immunomodulatory, pro-apoptotic, and autophagy-regulating effects.

For instance, several candidates may offer a novel therapeutic approach for APDS-associated intestinal diseases that respond poorly to rapamycin. These include dihydromyricetin, rhein, Shaoyao Decoction, Chang-An-Jun-Tai Decoction, and Xiang Lian Pill—all known for their potent anti-inflammatory and intestinal barrier-protective effects. Similarly, for pulmonary complications, agents including Andrographolide and Pingchuan Granules could alleviate respiratory symptoms through their anti-inflammatory and immunomodulatory activities. Regarding the elevated lymphoma risk, Prunella vulgaris extract and Compound Kushen Injection function to inhibit proliferation and induce apoptosis. They may therefore demonstrate superior efficacy over single-pathway inhibitors. Furthermore, the multi-component, multi-target mechanism inherent to Chinese herbal formulas presents a theoretically ideal strategy for complex diseases like APDS, where dysregulation of a single pathway leads to multi-system disorders. This poly-pharmacology has the potential to achieve synergy, concurrently correcting the underlying immune dysregulation and ameliorating clinical symptoms, thereby addressing both the origin and manifestations of the disease.

These prioritized TCM candidates offer a distinct mechanistic and therapeutic profile compared to rapamycin, the most commonly used targeted inhibitor in APDS. Rapamycin primarily inhibits mTORC1, partially controlling lymphoproliferation. However, it shows limited efficacy against APDS-associated enteropathy and does not directly target upstream PI3Kδ hyperactivation or broader immune dysregulation [8, 9]. Furthermore, its chronic use is associated with potential adverse effects, including metabolic abnormalities and increased susceptibility to infections due to broad immunosuppression. In comparison, candidates such as dihydromyricetin, rhein, and Shaoyao Decoction target multiple nodes within the PI3K/AKT/mTOR cascade, including upstream PI3K, central AKT, and downstream mTOR. In addition, they modulate intersecting pathways such as NF-κB and autophagy. This polypharmacological approach may offer a broader therapeutic window, potentially addressing both the root pathway hyperactivation and diverse clinical manifestations such as enteropathy and pulmonary complications with a more favorable safety profile. However, these hypotheses remain to be rigorously tested in APDS-specific models to determine whether this multi-target engagement translates into superior efficacy and tolerability over existing therapies.

Despite this therapeutic potential, the translational trajectory of these candidates is constrained by several pervasive limitations inherent in the current evidence base. A fundamental concern is that supporting data derive almost exclusively from indirect, non-APDS models, such as generic cancer cell lines or broad inflammatory models. As a result, the critical question of whether these candidates can rectify APDS-specific T and B cell dysfunction remains a fundamental gap in knowledge. Moreover, the mechanistic evidence remains largely observational. Most studies document changes in pathway phosphorylation without addressing the critical issue of PI3Kδ isoform selectivity or providing conclusive data from direct target engagement assays. Perhaps the most formidable challenge impeding clinical translation is the pervasive lack of standardization and reproducibility of TCM preparations. Our analysis reveals substantial heterogeneity in sourcing, processing, and extraction methodologies. This heterogeneity severely undermines the comparability of results across studies, confounds the accurate attribution of pharmacological effects, and jeopardizes the manufacturing consistency required for clinical development.

Advancing the field therefore demands a decisive shift toward direct functional validation and rigorous translational science. The core contribution of this review is to generate a prioritized list of testable hypotheses for future experimental validation, rather than to assert clinically ready efficacy. The most immediate imperative is to functionally validate the top-priority candidates in APDS-relevant immunological models. This requires testing them in primary immune cells from patients or genetically defined murine models. The key objective is to rigorously assess their capacity to reverse disease hallmarks such as pathway hyperactivation, abnormal proliferation, and cytokine dysregulation. In parallel, pharmacokinetic and safety studies must include a specific focus on TCM interactions with rapamycin or immunoglobulin replacement—a critical but unexplored area. Looking forward, integrating modern genotyping with the principles of TCM syndrome differentiation offers an innovative framework for personalizing therapy. Exploring potential “genotype–syndrome–formula” correlations could pave the way for tailored treatment strategies in this clinically heterogeneous patient population. In conclusion, this review provides a foundational and hypothesis-generating map of promising TCM-derived candidates for APDS. By framing our findings as a series of high-priority hypotheses, we aim to guide and stimulate rigorous investigation. The critical next step is to determine, through such investigation, whether these preclinical insights can be translated into tangible clinical benefits for patients.

## Supplementary Information

Below is the link to the electronic supplementary material.


Supplementary Material 1


## Data Availability

Not applicable.

## Abbreviations

APDS: Activated phosphoinositide 3 kinase δ syndrome
TCM: Traditional Chinese Medicine
PID: Primary Immunodeficiency Disease
IRT: Immunoglobulin Replacement Therapy
HSCT: Hematopoietic Stem Cell Transplantation
HLA: Human Leukocyte Antigen
PALR: Polysaccharides of Aconiti Lateralis Radix Praeparata
Sal A: Salvianolic acid A
Sal B: Salvianolic acid B
DHM: Dihydromyricetin
Amp: Ampelopsin
Cel: Celastrol
And: Andrographolide
TAAE: Toddalia asiatica alcohol extract
EGCG: Epigallocatechin-3-gallate
PVE: Prunella vulgaris extract
ZSD: Zi-Su-Zi decoction
C511: Compound 511
SYD: Shaoyao Decoction
CKI: Compound Kushen Injection
PCG: Pingchuan Granules
CAJT: Chang-An-Jun-Tai Decoction
XLP: Xiang Lian Pill
