# Supplementary material for: A review of PI3K/AKT/mTOR inhibitors from traditional Chinese medicine: potential and perspective for activated phosphoinositide 3-kinase δ syndrome
Source: Orphanet J Rare Dis. 2026 Jun 16;21:311. doi: 10.1186/s13023-026-04437-9 (PMC13536655; doi:10.1186/s13023-026-04437-9)
Supplement: Supplementary file 1 [file 13023_2026_4437_MOESM1_ESM.docx]

**Supplementary Table 1.** Search Strategy from Database Inception to October, 2025

| **Database** | **Search term** | **Results** |
| --- | --- | --- |
| PubMed | #1 ("PI3K"[All Fields] AND "mTOR"[All Fields]) OR "PI3K/AKT/mTOR"[All Fields]  #2 "herbal medicine"[MeSH Terms] OR ("herbal"[All Fields] AND "medicine"[All Fields]) OR "herbal medicine"[All Fields] OR "herbalism"[All Fields] OR "herbal"[All Fields] OR "herbals"[All Fields]  #1 AND #2 | 395 |
| CNKI | ( SU='PI3K/AKT/mTOR' OR (SU='PI3K' AND SU='mTOR') OR (SU='phosphatidylinositol 3-kinase' AND SU='mechanistic target of rapamycin') ) AND ( SU='traditional Chinese medicine' OR SU='Chinese herbal medicine' OR SU='herbal medicine' OR SU='natural medicine' OR SU='medicinal plants' OR SU='Chinese herbal compound' OR SU='Chinese herbal formula' OR SU='Chinese herbal extract' OR SU='Chinese herbal constituent' OR SU='traditional Chinese medicine' )* | 242 |
| Wanfang | ( Subject:("PI3K/AKT/mTOR") OR ( Subject:("PI3K") AND Subject:("mTOR") ) OR ( Subject:("phosphatidylinositol 3-kinase") AND Subject:("mechanistic target of rapamycin") ) ) AND ( Subject:("traditional Chinese medicine") OR Subject:("Chinese herbal medicine") OR Subject:("herbal medicine") OR Subject:("natural medicine") OR Subject:("medicinal plants") OR Subject:("Chinese herbal compound") OR Subject:("Chinese herbal formula") OR Subject:("Chinese herbal extract") OR Subject:("Chinese herbal constituent") )* | 554 |
| **TOTAL** | | 1191 |

*The actual search terms used in CNKI and Wanfang databases were in Chinese; the English translations are provided here for clarity.

**Supplementary Table 2. Herbs as Inhibitors: Characteristics and Experimental Details**

| Name | Target | Function | Reference |
| --- | --- | --- | --- |
| Sanguisorbae Radix | p-PI3K↓, p-AKT↓, p-mTOR↓, ROS↑, cleaved PARP-1↑, cleaved caspase-3↑ | Proliferation ↓, migration ↓, invasion ↓, apoptosis ↑, tumor growth inhibition ↓ | [20] |
| Radix Astragali fermented with Paecilomyces cicadae | PTEN↑, p-PI3K↓, p-AKT↓, p-mTOR↓, LC3-II/I↑, Beclin1↑, p62↓ | Autophagy↑, podocyte protection↑, apoptosis↓, oxidative stress↓ | [21] |
| Aconitum pendulum Busch | TNF-α↓, IL-6↓, IL-1β↓, PI3K↓, mTOR↓, HIF-1α↓, PGC-1α↑ | Inflammation ↓, apoptosis ↑, glycolysis ↓ | [22] |
| Orthosiphon stamineus Benth. | p-PI3K/PI3K↓, p-AKT/AKT↓, p-mTOR/mTOR↓ | Inflammation ↓, epithelial-mesenchymal transition ↓, renal interstitial fibrosis ↓ | [23] |
| Ainsliaea fragrans Champ. | COX-2↓, IDO1↓, FLG↑, TLR4↓, p-PI3K↓, p-AKT↓, mTOR↓ | Inflammation ↓ | [24] |

**Supplementary Table 3. Flavonoid Extracts from TCM as Inhibitors: Characteristics and Experimental Details**

| Name of inhibitor | Source | Method/source of extraction | Experimental dosages | Target | Function | Reference |
| --- | --- | --- | --- | --- | --- | --- |
| Myricetin | Bark and leaves of Myrica rubra | Purchased from Sigma-Aldrich | 0, 15, 25 μM | p-PI3K↓, p-AKT↓, p-mTOR↓, Bcl-2↓, Bax↑, Cleaved-PARP↑, Beclin-1↑, LC3-II/LC3-I↑ | Proliferation ↓, apoptosis ↑, autophagy ↑ | [25] |
| Baicalein | Scutellaria baicalensis | Purchased from Sigma-Aldrich | 0, 10, 20, and 40 μM | p-AKT↓, p-mTOR↓, NF-κB↓, p-IκB↓, IκB↑, Bcl-2↓, Bax↑, BECN1↑, LC3↑ | Proliferation ↓, apoptosis ↑, autophagy ↑ | [26] |
| Quercetin | Vegetables and Fruits | Purchased from China National Institutes for Food and Drug Control (content 99.1% by HPLC and 99.8% by UV) | 0, 12.5,50, and 200 μM | p-PI3K↓, p-AKT↓, p-DNA-PKcs↓, KU70↓, KU80↓, p-ATR↓, p-ATM↓, p-Chek1↓, p-Chek2↓, p-CDK1↑, Caspase-3↑, Bax↑, Bcl-2↓ | Proliferation ↓, apoptosis ↑ | [27] |
| Kaempferol | Numerous herbal medicines and fruits | -  (Only show abstract) | 0, 10, 20, 40 μM | p-PI3K↓, p-AKT↓, p-mTOR↓ | Proliferation ↓, migration ↓, apoptosis ↑, autophagy ↑ | [28] |
| Isorhamnetin | Chinese medicine and fruit, such as Hippophae rhamnoides L. | Purchased from Chromadex (Irvine, CA, USA), dissolved in dimethylsulfoxide (DMSO) and diluted to 20 mmol/l | 0, 10, 20, 40, 80 μM | p-AKT (Ser473)↓, p-p70S6K↓, p-4E-BP1↓, Cyclin B1↑ | Hyperplasia ↓ | [29] |
| Fisetin | Several fruits and vegetables such as strawberries, apples, persimmons, grapes, onions, and cucumbers | Purchased from MedChemExpress (MCE, Shanghai, China) (CAS No. : 528-48-3, purity of 98.02%) | 0, 50 and 100μM | PI3K↓, p-AKT↓, p-mTOR↓ | Proliferation ↓, migration ↓, invasion ↓, apoptosis ↑ | [30] |
| Apigenin | Fruits and vegetables | Purchased from Sigma-Aldrich | 0, 10, 20 and 40 μM | p-PI3K↓, p-AKT↓, p-mTOR↓, LC3-II↑, Beclin-1↑, Bcl-2↓ | Proliferation ↓, apoptosis ↑, autophagy ↑ | [31] |
| Nobiletin | Citrus fruits such as Citrus depressa and Citrus reticulate | Purchased from Chengdu Must Bio-Technology Co., Ltd. (MUST-22100917, Chengdu, China) (NOB, purity ≥ 98 %) | 0, 50, 100 and 200 μM | p-PI3K↓, p-AKT↓, p-mTOR↓, Beclin1↑, LC3II/LC3I↑ | Proliferation ↓, apoptosis ↑, autophagy ↑ | [32] |
| Lupiwighteone | Glycyrrhiza glabra | Purchased from Wuhan ChemFaces Biochemical Co., Ltd. (Wuhan, Hubei, China) | 10, 20, and 40 μM | PI3K↓, p-AKT↓, p-mTOR↓, Bcl-2↓ | Proliferation ↓, apoptosis ↑ | [33] |
| Delphinidin | Red wine and Berries | -  (Only show abstract and figures) | 0, 0.1, 1 and 10 μM | p-AKT↓, p-P70S6K↓, p-S6↓, p-ERK1/2↓, p-p38↓ | Proliferation ↓, apoptosis ↑ | [34] |
| Pelargonidin | Strawberries, Blueberries, Red radishes et al | No mentioned | 5 and 10 μM | p-PI3K↓, p-AKT↓, p-mTOR↓, VEGF↓, MMP2↓, MMP9↓, N-cadherin↓ | Proliferation ↓, migration ↓, apoptosis ↑, autophagy ↑ | [35] |
| Genistein | Soybean | Purchased from Sigma-Aldrich | 50 μM | p-PI3K↓, p-AKT↓, p-mTOR↓, NF-κB↓, Bcl-2↓, Bcl-xL↓, Bax↑ | Proliferation ↓, apoptosis ↑ | [36] |
| Brusatol | Bruceae Fructus | Purchased from Tauto Biotech (Shanghai, China) | 0 nM, 8 nM, 20 nM, 32 nM | p-PI3K↓, p-AKT↓, p-mTOR↓, Beclin-1↑, LC3-II/LC3-I↑, Bcl-2↓ | Proliferation ↓, migration ↓, apoptosis ↑, autophagy ↑ | [37] |
| Hesperidin | Citrus fruits | Purchased from Sigma‐Aldrich | 200 mg/kg/day for 16 weeks | PI3K↓, p-AKT↓, CDK2↓, Caspase-3↑ | Proliferation ↓, apoptosis ↑, antioxidant | [38] |
| Genkwanin | Genkwa Flos | -  (Only show abstract and figures) | 0, 20, 40 and 80 μM | PI3Kγ-p110↓, p-PI3K↓, p-AKT↓, p-mTOR↓, p-p70S6K↓, p-ULK↓, Bcl-2↓, Bcl-xL↓, p53↑, LC3-II↑, p62↓, ATG5↑ | Proliferation ↓, apoptosis ↑, autophagy ↑ | [39] |
| Pectolinarigenin | Citrus fruits | -  (Only show abstract and figures) | 0, 50 and 100 μM | p-PI3K↓, p-AKT↓, p-mTOR↓, p-p70S6K↓, Beclin-1↓, LC3-II/LC3-I↑ | Proliferation ↓, apoptosis ↑, autophagy ↑ | [40] |
| Silibinin | Dried fruits of silymarin | -  (Only show abstract and figures) | 0, 320, 370 and 420 μM | PIK3C2A↓, AKT-1↓, PTEN↓, mTOR↓, p-mTOR↓ | Hyperplasia ↓ | [41] |
| Synthesized silymarin-mediated | Plant Silybum marianum L. | Synthesized by mixing a silymarin solution with sodium selenite, followed by magnetic stirring and dialysis purification | 0, 0.4, 0.8, 1.6, 3.2, 6.4, 12.8, 25.6 μg/ml | p-PI3K↓, p-AKT↓, p-p70S6K↓, Bcl-2↓, LC3-II↑, p62↓, Beclin1↑ | Proliferation ↓, apoptosis ↑, autophagy ↑ | [42] |
| Isoorientin | Several plants such as lotus seed and honeysuckle | Purchased from Forever Biotechnology, Ltd. (Shanghai, China) (purity of ≥98%) | 0, 20, 40 and 80 μM | p-AKT↓, Bcl-2↓, LC3-II↑, Beclin1↑ | Proliferation ↓, apoptosis ↑, autophagy ↑ | [43] |
| Total flavonoid aglycones extract in Radix Scutellariae | Radix Scutellariae | Enzyme-assisted ethyl acetate extraction from Radix Scutellariae, standardized to 62.9% | 0, 3.2, 6.4, 12.8 μg/mL | p-PI3K↓, p-AKT↓, p-mTOR↓, p-p70S6K↓, LC3-II↑ | Proliferation ↓, apoptosis ↑, autophagy ↑ | [44] |
| Luteoloside | Gentianae Macrophyllae Radix | Isolated and purified from the medicinal plant Gentiana macrophylla. | 0, 10, 30, 60 µM | p-AKT↓, p-mTOR↓, p-p70S6K↓, LC3-II↑, Beclin1↑ | Proliferation ↓, autophagy ↑ | [45] |
| Quercetin-3-methyl ether | Several plants | Purchased from Sigma-Aldrich | 0, 1, 2.5, 5, 10 μM | AKT↓, p-mTOR↓, p-p70S6K↓ | Proliferation ↓, inflammation ↓ | [46] |
| Farrerol | Man-shan-hong [the dried leaves of Rhododendron dauricum L. (Ericaceae)] | Purchased from Dalian meilun biology technology co., LTD (Dalian, Liaoning, China) ( ≥98%, purity by HPLC) | 0, 12.5, 25 and 50 μM | p-PI3K↓, p-AKT↓, NF-κB↓ | Inflammation ↓ | [47] |
| Ampelopsin | Ampelopsis grossedentata | Purchased from Natural Ingredient, Inc., Guangxi, China | 0, 10, 25, 50, and 100 μM | p-AKT↓, NF-κB↓ | Inflammation ↓ | [48] |
| Oroxin B | Oroxylum indicum (L.) Vent | HY-N1435, purity: 99.71%, CAS number: 114482-86-9 | 0.2, 0.4, 0.6 mg/mL | p-PI3K↓, p-AKT↓, p-mTOR↓, Beclin-1↑, LC3 II/I↑ | Inflammation ↓, autophagy ↑ | [49] |
| Oroxylin A | Scutellariae Radix | Extracted and purified from the root of Scutellaria baicalensis | 0 μM, 4 μM, 8 μM, 16 μM | p-AKT↓, p-PI3K↓, p-ERK↓ | Inflammation ↓ | [50] |
| Pogostone | Pogostemonis Herba | Extracted from dried leaves of Pogostemon cablin by water-steam distillation, followed by alkaline extraction, acidification, ether partitioning, and recrystallization with n-hexane | 0, 25, 50, 100 μg/ml | p-AKT↓, p-mTOR↓, LC3-II↑, cleaved caspase-3↑, cleaved caspase-7↑ | Autophagy ↑, apoptosis ↑, proliferation ↓ | [51] |
| Calycosin | Astragalus membranaceus | Purchased from Yuanye Bio (Yuanye, Shanghai, China) | 0, 100, 200 and 400 μM | p-PI3K↓, p-AKT↓ | Inflammation ↓ | [52] |
| Cyanidin-3-glucoside | Fruits and Vegetables | Purchased from Tokiwa Phytochemical Co., Ltd., Japan (98.0% purity) | 10, 20, and 40 μM | p-PI3K↓, p-AKT↓, p-mTOR↓, Bcl-2↓ | Migration ↓, invasion ↓, proliferation ↓, apoptosis ↑ | [53] |
| Puerarin | Puerariae Lobatae Radix | Purchased from Shanghai Aladdin Biochemical Technology Co., Ltd., purity 98%, as a monomeric compound | 0, 0.0625, 0.125, 0.25, 0.5, 1.0, 2.0, 4.0, 6.0, 8.0, 16.0 mmol/L | PI3K↓, P-PI3K↓, AKT↓, P-AKT↓, mTOR↓, LC3B↑, P62↓, BCL2↓, AMPK↓, | Proliferation ↓, autophagy ↑, apoptosis ↑ | [54] |
| Dihydromyricetin | Wild vines | Purchased from Shanghai Yuanye Bio-Technology Co., Ltd., China (CAS: 27200-12-0, purity ≥98%) | 0, 12.5, 25, 50, 100 μM | p-PI3K↓, p-AKT↓, p-mTOR↓, LC3A/B↑ | Inflammation ↓, Fibrosis ↓, Autophagy ↑ | [55] |

**Supplementary Table 4. Terpenoid Extracts from TCM as Inhibitors: Characteristics and Experimental Details**

| Name of inhibitor | Source | Method/source of extraction | Experimental dosages | Target | Function | Reference |
| --- | --- | --- | --- | --- | --- | --- |
| Bruceine D | The shrub Brucea javanica (L.) Merr. (Simaroubaceae) | Provided by the Institute of Traditional Chinese Medicine and Natural Products, Jinan University (≥98% purity) | 0, 1, 2, 4 μM | PI3K↓, p-AKT↓ | Proliferation ↓, migration ↓, invasion ↓ | [56] |
| Betulinic acid | The bark of the white birch tree (Betula pubescens) | Purchased from Sigma‐Aldrich | 0, 10, 20, 40 μM | p-AKT↓, p-mTOR↓, NF-κB↓, Bcl-2↓, LC3B↑ | Proliferation ↓, apoptosis ↑, autophagy ↑ | [57] |
| Stigmasterol | Achyranthes bidentata | Purchased from Sigma‐Aldrich | 1 and 5 μM | p-AKT↓, TNF-α↓, VEGFR-2↓, p-Src↓, p-PCL↓, p-FAK↓ | Inflammation ↓, angiogenesis ↓ | [58] |
| Jolkinolide B | Euphorbia fischeriana Steud | Purchased from Must Bio-Technology Co. (molecular weight of 330.4, purity > 99%) | 60μg/mL | PTEN↑, p-PI3K↓, p-AKT↓, Bcl-2↓, Bax↑ | Proliferation ↓, migration ↓, invasion ↓, apoptosis ↑ | [59] |
| Afrocyclamin A | Androsace umbellata | Isolated from Androsace umbellata via methanol extraction, n-butanol fractionation, and C18 column chromatography | 0, 1, 4, 8 μM | p-PI3K↓, p-AKT↓, p-mTOR↓, Bcl-2↓, LC3-II↑, | Proliferation ↓, apoptosis ↑, autophagy ↑, migration ↓, invasion ↓ | [60] |
| Celastrol | Tripterygium wilfordii Hook F (Lei Gong Teng） | Purchased from Solarbio Science & Technology Co., Ltd (purity ≥ 98%, 909D021) | 0.5, 1 and 2 mg/kg | p-PI3K↓, p-AKT↓, p-mTOR↓, LC3-II/LC3-I↑, Beclin-1↑ | Proliferation ↓, autophagy ↑, inflammation ↓ | [61] |
| Ganoderma tsugae | Ganoderma | Ethanol extraction of Ganoderma fruiting body powder, concentrated under reduced pressure. | 0, 200, 300, 400 µg/mL | p-PI3K↓, p-AKT↓, p-mTOR↓, Bcl-2↓, Beclin-1↑, LC3-II↑ | Proliferation ↓, apoptosis ↑, autophagy ↑ | [62] |
| Ursolic acid | Apples, Hawthorn, Cranberries, and many Chinese medicinal plants, such as Hedyotis diffusa, Cornus officinalis, and Ligustrum lucidum | Naturally occurring triterpenoid acid isolated from plant waxes and herbs | 0, 6, 12, 24 μM | p-PI3K↓, PI3K↓, p-AKT↓, AKT↓ | Proliferation ↓, apoptosis ↑ | [63] |
| Demethylzeylasteral | The entire plant of Tripterygium wilfordii Hook F or peeled wood parts | Purchased from Target Molecule Corp | 1, 3 and 10 μM | p-AKT↓, Bcl-2↓ | Proliferation ↓, apoptosis ↑, migration ↓ | [64] |
| Germacrone | Rhizoma curcuma | No mentioned | 0, 40, 80 and 160 μM | p-PI3K↓, p-AKT↓, p-mTOR↓ | Proliferation ↓, apoptosis ↑, migration ↓ | [65] |
| Helenalin | Centipeda minima | No mentioned | 0.25, 0.5 mg/kg | p-PI3K↓, p-AKT↓, p-mTOR↓, p-P70S6K↓, PTEN↑ | Fibrosis ↓, inflammation ↓ | [66] |
| Andrographolide | Andrographis paniculata | Purchased from Solarbio | 0, 5, 10, 20 mg/kg | p-PI3K↓, AKT↓, p-mTOR↓ | Autophagy ↑, inflammation ↓ | [67] |
| Beta-Elemene | Rhizoma zeodaria | No mentioned | 3 μg/mL | p-PI3K↓, p-AKT↓, p-mTOR↓, Bcl-2↓ | Apoptosis ↑ | [68] |
| An ethyl acetate fraction of Artemisia capillaris (ACE-63) | Artemisia capillaris | Obtained from the 70% ethanol extract of Artemisia capillaris aerial parts, subsequently purified by liquid-liquid fractionation and freeze-drying | 50, 100 μg/ml | p-AKT↓, p-mTOR↓, p-P70S6K↓ | Proliferation ↓, apoptosis ↑, migration ↓ | [69] |
| Safranal | Crocus sativus L. | Purchased from MedChemExpress (USA) | 0, 50, 100, 200, 400, 800 μM | PI3K↓, AKT↓, mTOR↓, p-AKT↓, p-mTOR↓, CDK1↓, CCNB1↓, CASP3↑, CASP7↑, PARP1↑ | Apoptosis ↑, proliferation ↓, invasion ↓, migration ↓ | [70] |
| TEEG (3β,16β,23-trihydroxy-13,28-epoxyurs-11-ene-3-O-β-D-glucopyranoside) | Chinese medicine formula Shenqi San (CE-SS) | No mentioned | 1, 2, 4, and 6 μg/ml | p-PI3K↓, p-AKT↓, p-mTOR↓, p-p70S6K↓ | Proliferation ↓, autophagy ↑ | [71] |
| Gypenosides | G. pentaphyllum | Extracted from Gynostemma pentaphyllum leaves using 80% ethanol via reflux extraction, followed by concentration under reduced pressure | 550 μg/mL and 180 μg/mL | p-PI3K↓, p-AKT↓, p-mTOR↓, Bcl-2↓ | Proliferation ↓, apoptosis ↑ | [72] |
| Ginsenoside Rg3 | Chinese medical herb, ginseng | Purchased by Yuanye Inc., China | Inhibit PI3K/AKT/mTOR | p-AKT↓, PI3K/AKT/mTOR↓ | Migration ↓, proliferation ↓, apoptosis ↑ | [73] |
| Astragaloside IV | Radix astragali | Purchased from Merck with a purity of ≥90% | 20 mg/(kg·d) | p-PI3K↓, p-AKT↓, p-mTOR↓ | Inflammation ↓ | [74] |
| Astragaloside II | Radix astragali | Purchased from Shanghai MCE Co., Ltd., China; commercially available purified compound isolated from total saponins of Astragalus membranaceus | 0, 5, 10, 20, 30, 40, 80 μM | p-PI3K↓, p-AKT↓, p-mTOR↓, Bcl-2↓ | P\Proliferation ↓, apoptosis ↑, migration ↓, invasion ↓ | [75] |
| Panax Notoginseng Saponins | Notoginseng Radix et Rhizoma | Purchased from Dalian Meilun Biotechnology Co., Ltd. (batch number: MB6091) | 100 mg/kg | AKT1↓, mTOR↓, p-PI3K↓, p-AKT↓, p-mTOR↓, P62↓, LC3-II/LC3-I↑ | Autophagy ↑, inflammation ↓ | [76] |

**Supplementary Table 5. Steroid Extracts from TCM as Inhibitors: Characteristics and Experimental Details**

| Name of inhibitor | Method/source of extraction | Experimental dosages | Target | Function | Reference |
| --- | --- | --- | --- | --- | --- |
| Rhizoma Paridis saponins | 70% Ethanol extraction, D101 resin (65% ethanol) purification, vacuum concentration. | 100 mg/kg/day | p-PI3K↓, p-AKT↓, NF-κB↓ | Invasion ↓, Inflammation ↓ | [77] |
| The saponin DT-13 | Provided as a powder (95% purity) by Tianjin Tasty Pharmaceutical Co., Ltd. | 0, 0.1, 1, 10 µM | p-AKT↓ | Migration ↓ | [78] |
| Bufalin | Purchased from Shanghai Yuanye Biological Technology Company; purity ≥98% | 0, 2.5, 5, 10, 20, 40, 80 nM | p-AKT↓, p-mTOR↓, PI3K↓ | Proliferation ↓, migration ↓, invasion ↓ | [79] |
| Polyphyllin VI | Extracted from Trillium tschonoskii with purity >99% | 2.5, 5.0, 7.5 mg/L | p-PI3K↓, p-AKT↓, p-mTOR↓, LC3B II/I↑ | Proliferation ↓, migration ↓, apoptosis ↑, autophagy ↑ | [80] |

**Supplementary Table 6. Alkaloid Extracts from TCM as Inhibitors: Characteristics and Experimental Details**

| Name of inhibitor | Source | Method/source of extraction | Experimental dosages | Target | Function | Reference |
| --- | --- | --- | --- | --- | --- | --- |
| Krukovine | Abuta grandifolia (Mart.) Sandw. (Menispermaceae) | Purchased from Specs (Bleiswijkseweg, The Netherlands) | 12.5, 25, 50 μM | p-PI3K↓, p-AKT↓, p-mTOR↓ | Migration ↓, invasion ↓, proliferation ↓, apoptosis ↑ | [81] |
| Tetrandrine | Stephania tetrandra S Moore | Purchased from the company of Hao-xuan Bio-tech (Xi'an, China) | 0, 1, 2, 4 μM | p-PTEN↓, p-AKT1/2/3↓, Bcl-2↓ | Proliferation ↓, apoptosis ↑ | [82] |
| Matrine | Sophora flavescens Ait | Purchased from Ningxia Zijinghua Pharmaceutical Co., LTD. | 0, 1.2, 2.4, 4.8 mM | p-AKT↓, p-mTOR↓, LC3-II↑ | Autophagy ↑, proliferation ↓, apoptosis ↑ | [83] |
| Oxymatrine | Chinese medical herb, ginseng | Purchased from Shanghai Aladdin Bio-Chem Technology Co., Ltd. | 0, 0.5, 1, 2 mg/ml | p-AKT↓, p-mTOR↓, Bcl-2↓ | Invasion ↓, proliferation ↓, apoptosis ↑ | [84] |
| Berberine | Roots, Rhizomes and Stem bark of a number of plants (for example, Berberis aristata, Berberis aquifolium and Berberis vulgaris) of the Berberidaceae family | Purchased from Sigma-Aldrich (St. Louis, MO) | 2.5, 5 μM | p-AKT↓, p-IKK↓, p-NF-κB↓ | Inflammation ↑, apoptosis ↑ | [85] |
| Alkaloids of Aconiti Lateralis Radix Praeparata | Aconiti Lateralis Radix Praeparata | Purchased from Bozhou Yonggang Pharmaceutical Co., Ltd (Sichuan, China) | 0, 50, 100, 200 μg/mL | p-PI3K↓, p-AKT↓ | Proliferation ↓, glycolysis ↓ | [86] |
| Toddalia asiatica alcohol extract | Toddalia asiatica(L.) Lam | Extracted from dried root bark using 70% ethanol under reflux at 85°C, followed by concentration, freezing, and freeze-drying to obtain TAAE powder | 0, 0.25, 0.5, 1 mg/mL | p-PI3K↓, p-AKT↓, p-mTOR↓, LC3-II/I↑, Beclin1↑, Atg5↑, p62↓, TNF-α↓, IL-6↓, COX-2↓ | Autophagy↑, inflammation↓, macrophage activation↓, immune regulation | [87] |
| Brucine | Strychni Semen | Purchased from Carl Roth GmbH, Germany | 0.5 mmol/L | PI3K↓, AKT↓, mTOR↓, LC3-II↑, Beclin-1↑ | Proliferation ↓, migration ↓, autophagy ↑ | [88] |

**Supplementary Table 7. Phenol Extracts from TCM as Inhibitors: Characteristics and Experimental Details**

| Name of inhibitor | Source | Method/source of extraction | Experimental dosages | Target | Function | Reference |
| --- | --- | --- | --- | --- | --- | --- |
| Deoxypodophyllotoxin | Anthriscus sylvestris | Isolated from Anthriscus sylvestris roots | 0, 10, 20, 40 nM | p-AKT↓, p-mTOR↓, LC3B-II↑ | Proliferation ↓, apoptosis ↑, autophagy ↑ | [89] |
| Rosemary extract | Rosmarinus officinalis plant | Sequential extraction of dried leaves with dichloromethane:methanol (1:1) and methanol, followed by solvent removal via rotary evaporation | 0, 25, 50 μg/mL | p-AKT↓, AKT↓, p-mTOR↓, mTOR↓, p-p70S6K↓, p70S6K↓ | Proliferation ↑, apoptosis ↑ | [90] |
| Quercetin-3-O-glucoside | Plum fruit polyphenols | Purchased from Sigma-Aldrich (St. Louis, MO) | 0, 50, 100 nM | p-AKT↓ | Migration ↓ | [91] |
| Licochalcone A | Glycyrrhiza glabra (licorice) and Glycyrrhiza inflata (Chinese licorice) | Purchased from Sigma-Aldrich, Merck KGaA | 0, 10, 20, 50 μM | p-AKT↓, p-mTOR↓, LC3-II↑, Bcl-2↓ | Proliferation ↑, autophagy ↑, apoptosis ↑ | [92] |
| Curcumin | Curcuma longa plant | Purchased from Sigma-Aldrich (St. Louis, MO) | 58 mg/kg | PI3K↑, AKT↑, AMPK↑, mTOR↑ | Fatigue ↓ | [93] |
| Pterostilbene | Blueberries, Tree wood, and several varieties of grapes | No mentioned | 0, 1.25, 2.5, 5, 10, 20, 40, 80 μmol/L | p-PI3K↓, p-AKT↓, PI3K↓, AKT↓ | Proliferation ↓, apoptosis ↑, migration ↓, invasion ↓ | [94] |
| Epigallocatechin-3-gallate (EGCG) | Green tea | No mentioned | 0, 5, 10, 20, 40, 80 μM | p-PI3K↓, p-AKT↓ | Proliferation ↓, invasion ↓, migration ↓, apoptosis ↑, inflammation ↓ | [95] |
| Osthole (7-methoxy-8-isopentenoxycoumarin, C15H16O3) | Apiaceae family such as Cnidium monnieri (L.) Cusson and Angelica pubescens Maxin. f. and Peucedanum ostruthium (L.). | Purchased from Solarbio Science & Technology (Beijing, China) | 0, 40, 50, 80, 100, 120, 150, 160, 200, 240 μM | PI3K↓, AKT↓, mTOR↓, Bcl-2↓ | Proliferation ↓, apoptosis ↑ | [96] |
| Resveratrol (3,4,5-trihydroxystilbene) | Polygonum cuspidatum, red wine and the red grape skins | Purchased from Sigma Chemical Co. (St. Louis, MO, USA) | 150–200 μM | PTEN↑, AKT↓, mTOR↓, Bcl-2↓ | Proliferation ↓, apoptosis ↑ | [97] |
| 1,2,3,4,6-penta-O-galloyl-beta-D-glucose (PGG) | Many herbs, such as Rhus chinensis MILL and Paeonia suffruticos | PGG was isolated from the gallnut methanol extract through sequential solvent fractionation, silica gel column chromatography, and final purification by HPLC. | 0, 25, 50 μM | PI3K↓, p-AKT↓, p-mTOR↓ | Angiogenesis ↓ | [98] |
| Ellagic acid (EA, 4,4′,5,5′,6,6′-hexahydroxydiphenic acid 2,6,2′,6′-dilactone) | Berries and Nuts | Purchased from Yuanye Biotech, Shanghai, China | 0, 40, 80 μM | p-AKT↓ | Proliferation ↓, migration ↓, invasion ↓ | [99] |
| Salidroside | Rhodiolae Crenulatae Radix et Rhizoma | Purchased from Nanjing Gerui Biotechnology Co., Ltd. | 40 μmol/L | p-PI3K↓, p-AKT↓, p-mTOR↓, LC3B II/I↑, Beclin-1↑, Bcl-2↓ | Autophagy↑, Apoptosis↑, proliferation↓ | [100] |

**Supplementary Table 8. Quinone Extracts from TCM as Inhibitors: Characteristics and Experimental Details**

| Name of inhibitor | Source | Method/source of extraction | Experimental dosages | Target | Function | Reference |
| --- | --- | --- | --- | --- | --- | --- |
| Salvianolic acid A(Sal A) | Salvia | Purchased from Solarbio | 0, 25, 50, 100 μM | TAGLN2↓, p-PI3K↓, p-AKT↓ | Proliferation ↓, migration ↓, invasion ↓, apoptosis ↑ | [101] |
| Salvianolic acid B (Sal B) | Salvia | No mentioned | 0, 25, 50 μg/mL | p-AKT↓, AKT↓, p-mTOR↓, mTOR↓, p-p70S6K↓, p70S6K↓ | Proliferation ↓, apoptosis ↑ | [102] |
| Mollugin | R. cordifolia L | Purchased from the National Institute for the Control of Pharmaceutical and Biological Products (Beijing, China) | 0, 12, 24, 36 μM | p-AKT↓, PKM2↓, Bcl-2↓ | Proliferation ↓, apoptosis ↑ | [103] |
| Shikonin | Lithospermum erythrorhizon | Extracted from the roots of Lithospermum erythrorhizon using organic solvents like ethanol or can be purchased from chemical suppliers such as Sigma-Aldrich | 0, 10, 20, 50 μM | p-PI3K ↓, p-AKT ↓, p-mTOR ↓, LC3-II ↑, Bcl-2 ↓ | Proliferation ↓, autophagy ↑, apoptosis ↑ | [104] |
| Coenzyme Q0 | Antrodia camphorata | Prepared from broth in water, filtered, and isolated by HPLC (RP-18 column, acetonitrile/0.05% TFA gradient, 254/220 nm detection) | 0, 10, 20, 30 μM | p-PI3K ↓, p-AKT ↓, p-mTOR ↓, LC3-II ↑, Bcl-2 ↓ | Proliferation ↓, autophagy ↑, apoptosis ↑ | [105] |
| Tanshinone IIA | Salvia | Purchased from the Zhejiang Institute for Drug Control (Hangzhou, Zhejiang, China) | 0, 1, 2, 4, 6 µM | p-AKT↓, PI3K↓ | Proliferation ↓, migration ↓ | [106] |
| Cryptotanshinone | Salvia | Purchased from Xi'an Helin Biological Engineering Co. (Xi-an, Shanxi, China) | 0, 0.625, 1.25, 2.5, 10, 20, 40, 80 μM | p-PI3K↓, p-AKT↓, p-mTOR↓, IL-4↓, IL-6↓, IL-18↓, TNF-α↓, IFN-γ↓, Pro-IL-8↓, Pro-IL-1β↓, NLRP3↓ | Proliferation ↓, invasion ↓, inflammation ↓ | [107] |
| Danshen injection | Salvia | Purchased from CHITAI QINGCHUNBAO PHARMACEUTICAL Co., Ltd. (Zhejiang, China) | p-AKT↓, PI3K↓, mTOR↓, LC3B-II↑, Beclin1↑ | Inhibit p-PI3Kp-AKT and p-mTOR | autophagy ↑ | [108] |
| Hydroxysafflor yellow A | safflower (Carthamus tinctoriusL.) | No mentioned | 5, 10, and 20 μM | PI3Kα (PIK3CA1)↓, p-PI3K↓, p-AKT↓, p-mTOR↓, p-NF-κB↓ | Oxidation ↓, inflammation ↓ | [109] |
| Emodin | Rhei Radix et Rhizoma | Purchased from Shanghai Xuehui Technology Co., Ltd. | 0, 10, 20, 40, 80 μmol/L | p-AKT↓, p-mTOR↓, p-NF-κB↓, Bcl-2↓ | Proliferation ↓, apoptosis ↑ | [110] |
| Rhein | Rhei Radix et Rhizoma | Purchased from Chengdu Dest Biological Technology Co., Ltd. | 0, 6.25, 12.5, 25, 50 μM | p-PI3K↓, p-AKT↓, p-mTOR↓, p-p70S6K↓ | Inflammation ↓ | [111] |

**Supplementary Table 9. Lignan Extracts from TCM as Inhibitors: Characteristics and Experimental Details**

| Name of inhibitor | Source | Method/source of extraction | Experimental dosages | Target | Function | Reference |
| --- | --- | --- | --- | --- | --- | --- |
| Deoxypodophyllotoxin (anthricin) | Anthriscus sylvestris (L.) Hoffm | isolated from Anthriscus sylvestris roots with structural confirmation by NMR | 0、10、20、40 nM | p-AKT↓, p-mTOR↓, LC3B-II↑ | Proliferation ↓, apoptosis ↑, autophagy ↑ | [112] |
| Honokiol | Magnoliae officinalis cortex (Hou Po) | No mentioned | 0, 5, 10, 20 μg/ml | p-PI3K↓, p-AKT↓, p-mTOR↓, LC3II↑ | Proliferation ↓, apoptosis ↑, autophagy ↑ | [113] |
| Schisandrin B | Schisandra Michx. | Purchased from Chengdu Herburify Co., Ltd. | 0, 20, 40, 60, 80 μM | p-AKT (Ser473)↓, p-AKT (Thr308)↓, Bcl-2↓ | Proliferation ↓, migration ↓, invasion ↓, apoptosis ↑ | [114] |

**Supplementary Table 10. Other or Unknown Extracts from TCM as Inhibitors: Characteristics and Experimental Details**

| Name of inhibitor | Source | Method/source of extraction | Experimental dosages | Target | Function | Reference |
| --- | --- | --- | --- | --- | --- | --- |
| Spatholobus suberectus Column Extract | Spatholobus suberectus | Purchased from Beijing Xinglin Pharmaceutical Co., Ltd. | 0, 80, 160, 320 μg/mL | p-PI3K↓, p-AKT↓, p-mTOR↓, Bcl-2↓ | Proliferation ↓, apoptosis ↑, migration ↓ | [115] |
| Celastrus orbiculatus ethyl acetate extracts | Celastrus orbiculatus | Purchased from Zhixin Pharmaceutical Co., Ltd. (Guangzhou, China) | 0, 20, 40, 80, 160, 320 μg/mL | p-AKT (Thr308)↓, p-mTOR (Ser2448)↓, p-p70S6K (Thr389)↓, LC3-II↑, Beclin-1↑, Bax↑, Bcl-2↓ | Proliferation ↓, apoptosis ↑, autophagy ↑ | [116] |
| Centipeda minima ethanol extracts | Centipeda minima | Prepared by macerating 30 g of powdered herb in 95% ethanol at room temperature for 72 hours, followed by filtration, evaporation at 40°C, and lyophilization, yielding 12.65% (3.80 g). | 0, 20, 40, 60, 80 µM | p-AKT (Ser473)↓, p-AKT (Thr308)↓, Bcl-2↓ | Proliferation ↓, migration ↓, invasion ↓, apoptosis ↑ | [117] |
| Zanthoxylum bungeanum seed oil | Edible drug Zanthoxylum bungeanum seeds | -  (Only show abstract) |  | PI3K↓, p-AKT↓, p-mTOR↓, LC3-II↑, Beclin-1↑ | Proliferation ↓, apoptosis ↑, autophagy ↑ | [118] |
| Sarcandra Glaba | Sarcandrae Herba | Purchased as Sarcandra Glabra capsules from Jilin Junhong Pharmaceutical Co., Ltd., China (Lot No.: 20090701) | 0.625, 1.25, 2.5 mg/mL | PI3K↓, AKT↓, mTOR↓, P70S6K↓ | Proliferation ↓ | [119] |
| Marsdenia tenacissima extract | Marsdenia tenacissima (Roxb.) Wight et Arn | A standardized water extract from the stem of Marsdenia tenacissima, further purified by ethanol precipitation and quality-controlled via HPLC and marker compounds (chlorogenic acid and tenacigenoside A). | 0, 5, 10, 20 g/kg | p-PI3K↓, p-AKT↓, p-mTOR↓ | Proliferation ↓, apoptosis ↑ | [120] |
| Kochia scoparia seed extract | Kochia scoparia (L.) Schrad (Amaranthaceae) | A methanol extract from Kochia scoparia seeds, prepared by room-temperature maceration, filtration, and rotary evaporation. | 0, 5, 10, 15, 20 µg/mL | p-PI3K↓, p-AKT↓, p-mTOR↓, Bcl-2↓ | Migration ↓, invasion ↓, proliferation ↓, apoptosis ↑, angiogenesis ↓ | [121] |
| Forsythia suspensa extract | Forsythia Suspensa | Purchased from Shuangpikayahong Protein Company (commercial source) | 0, 10, 15 μmol/L | p-PI3K↓, p-AKT↓, p-mTOR↓ | Proliferation ↓, apoptosis ↑ | [122] |
| Prunella vulgaris extract | Prunellae Spica | Obtained by alcohol decoction, extraction, distillation, and drying processes | 3.125, 6.25, 12.5, 25, 50, 100, 200 μg/ml | p-AKT↓, p-mTOR↓ | Proliferation ↓, apoptosis ↑ | [123] |

**Supplementary Table 11. Chinese Medicine Formulas as Inhibitors: Characteristics and Experimental Details**

| Name of inhibitor | Composition | Main ingredient that makes a difference | Method/source of extraction | Target | Function | Reference |
| --- | --- | --- | --- | --- | --- | --- |
| The Qingyihuaji decoction | Scutellaria barbata D.Don 30g, Gynostemma pentaphyllum (Thunb.) Makino 15g, Oldenlandia diffusa (Willd.) Roxb. 15g, Ganoderma lucidum (Leyss. ex Fr.) Karst. 15g, Myristica fragrans Houtt. 5g, Amorphophallus kiusianus (Makino) Makino 15g, and Coix lacryma-jobi var. ma-yuen (Rom.Caill.) Stapf 15g | Quercetin, luteolin, wogonin, beta-sitosterol, and baicalein | Prepared by mixing individual herb granules, each produced through water immersion, double boiling, mixing and condensing decoctions into a thick paste, followed by reduced-pressure drying and pulverization | p-PI3K↓, p-AKT↓, p-mTOR↓, Bcl-2↓ | Proliferation ↓, migration ↓, invasion ↓, apoptosis ↑, inflammation ↓ | [124] |
| Sijunzi Decoction | Panax ginseng, C.A.Mey., Atractylodes macrocephala Koidz., Poria cocos (Schw.) Wolf., and Glycyrrhiza uralensis Fisch. | Kaempferol, Cerevisterol, Jaranol, Isorhamnetin, 5-(2,2-Difluorovinyl)-2′-deoxyuridine, Medicarpin, 7-Methoxy-2-methyl isoflavone, Glyasperin B, Isotrifoliol, (E)-1-(2,4-dihydroxyphenyl)-3-(2,2-dimethylchromen-6-yl)prop-2-en-1-one | Prepared by double water decoction (10x and 8x water, boiled for 40 and 30 min), followed by filtration, concentration, and freeze-drying to yield a powder | p-PI3K↓, p-AKT↓, p-mTOR↓, Bcl-2↓, LC3-II/I↑, Beclin-1↑ | Proliferation ↓, apoptosis ↑, autophagy ↑ | [125] |
| Wei-Tong-Xin | Rheum offcinale Baill., Pharbitis nil (L.) Choisy., Aucklandia lappa Decne., Gleditsia sinensis Lam. and Glycyrrhiza uralensis Fisch | Rheum offcinale Baill (ROB), Pharbitis nil (L.). Choisy. (PNC), Aucklandia lappa Decne. (ALD), Gleditsia sinensis Lam. (GSL)and Glycyrrhiza uralensis Fisch. (GUF). | Extracted from crushed herbs by triple reflux with 90% ethanol, followed by concentration, oven-drying at 60°C, and freeze-drying at -70°C to obtain a powder | p-PI3K↓, p-AKT↓, p-mTOR↓, Bcl-2↓ | Proliferation ↓, migration ↓, invasion ↓, apoptosis ↑ | [126] |
| Fuzheng Jiedu Decoction | Pseudostellaria heterophylla (Miq.) Pax 15g, Atractylodes macrocephala Koidz 10g, Poria cocos (Schw.) Wolf 10g, Actinidia chinensis Planch 15g, and Polygonum cuspidatum 15g | - | Prepared by mixing five herbs, soaking in 500 ml distilled water for 30 min, boiling and extracting twice, and concentrating to 0.262, 0.524, and 1.048 g/ml solutions stored at -4°C | p-PI3K↓, p-AKT↓, p-mTOR↓, NF-κB↓, p38↓ | Proliferation ↓, apoptosis ↑, invasion ↓ | [127] |
| modified Jianpi Yangzheng decoction | Astragali Radix, the root of Astragalus membranaceus (Fisch.) 60 g, Codonopsis Radix, root of Codonopsis pilosula (Franch.) 30 g, Sparganii rhizoma, root of Sparganium stoloniferum (Buch.-Ham. ex Graebn.) 30 g and Curcumae Rhizoma, root of Curcuma zedoaria (Christm.) 30 g | Rutin, lobetyolin, calycosin-7-glucoside, formononetin, calycosin, ononin and p-coumaric acid | Prepared by concentration to 1.0 g/ml, sterile filtration (0.22 μm), and storage at 4°C | p-PI3K↓, p-AKT↓, p-mTOR↓,Bcl-2↓ | Proliferation ↓, apoptosis ↑ | [128] |
| Weipixiao decoction | Astragalus, Radix Pseudostellariae, Atractylodes macrocephala, Salvia miltiorrhiza Bge, Oldenlandia diffusa(Willd.) Roxb | Calycosin-7-glucoside, ginsenoside-Rg1, ginsenoside-Rb1, astragaloside IV, atractylenolide III, II, and I, | No mentioned | PI3K↓, AKT↓, mTOR↓ | Proliferation ↓ | [129] |
| Kanglaite® injection | No mentioned | No mentioned | Purchased from Zhejiang Kanglaite Pharmaceutical Co., Ltd (Hangzhou, China) | p-AKT↓, p-mTOR↓ | Proliferation ↓, apoptosis ↑ | [130] |
| Gubenyiliu formula II | Ligusticum chuanxiong Hort., Millettia reticuiata Benth, and Curcuma zedoaria (Christm.) Rosc, Sarcandra glabra (Thunb.) Nakai, Fritillaria thunbergii Miq, Sophora flavescens Alt, Codonopsis pilosula (Franch.) Nannf, Poria cocos (Schw.) Wolf, Atractylodes macrocephala Koidz, Astragalus mongholicus Bunge, Fructus Ligustri Lucidi, Lycium barbarum L., Epimedium brevicornu Maxim | No mentioned | Extracted by decocting twice with 10 volumes of water for 45 min each time, then concentrating the combined decoction to 1.5, 0.375 and 0.825 g/ml, and storing it at 4°C. | p-PI3K↓, p-AKT↓, p-mTOR↓,Beclin1↑,LC3-II/I↑ | Proliferation ↓, migration ↓, invasion ↓, apoptosis ↑, autophagy ↑ | [131] |
| NPC01 | Glycyrrhizae Radix Preparata 60g, Rheum palmatum L. 60g, Ligusticum chuanxiong Hort 75g, Coptis chinensis Franch., and Forsythia suspensa 120g | No mentioned | Prepared by extracting a mixture of five medicinal herbs in a standard ratio with purified water via reflux at 100°C for 2 h, followed by filtration, lyophilization, and storage at −80°C | p-AKT↓, p-mTOR↓, HIF-1α↓, VEGF↓ | Proliferation ↓, apoptosis ↑ | [132] |
| CFF-1 | Radix Aconiti Lateralis Preparata, Poria, Ramulus Cinnamomi, Radix Rehmanniae Preparta, Rhizoma Polygonati, Rhizoma Curcumae, Herba Polygoni Perfoliati, Rhizoma Wenyujin Concisum, Paris polyphylla Smith, Radix Cyathulae, and Radix Glycyrrhizae, etc | No mentioned | Extracted from smashed Traditional Chinese Medicine materials by soaking in absolute ethanol, followed by centrifugation, and the supernatant was concentrated to 1 g/ml via low-temperature evaporation | p-PI3K↓, p-AKT (Ser473, Thr308)↓, p-mTOR (Ser2448)↓, p-p70S6K↓, Bcl-2↓, Beclin-1↑, LC3-II↑ | Proliferation ↓, migration ↓, invasion ↓, apoptosis ↑, autophagy ↑ | [133] |
| NSENL | Ginseng (2 parts), milkvetch root (3 parts), glehnia (2 parts), ophiopogon (2 parts), polygonatum odoratum (1 part), Stephania tetrandra (1 part), hedyotis (1 part), and pseudo-ginseng granules (1 part) | Ginsenoside Rg1, ginsenoside Rb1, ginsenoside Rg3, astragaloside IV, ophiopogonin D, and tetrandrine | Dissolved in 150 ml methanol, ultrasonically extracted for 2 hours, filtered, and adjusted to volume | p-AKT↓, p-mTOR↓ | Reversing multidrug resistance | [134] |
| Xihuang Pill | Calculus Bovis, Moschus, Boswellia frereana, and Commiphora myrrha | Valine, 4-gingerol, myrrhone, ricinoleic acid, glycocholic acid, curzerenone, 11-keto-β-boswellic acid, oleic acid, germacrone, 3-acetyl-9,11-dehydro-β-boswellic acid, 5β-androstane-3,17-dione, and 3-acetyl-11-keto-β-boswellic acid | Extracted by soaking in water for 24 h, ultrasonic dissolution, centrifugation, and resuspension in DMSO | p-PI3K↓, p-AKT↓, p-mTOR↓ | Proliferation↓, migration↓, apoptosis↑ | [135] |
| Guizhi Fuling Wan | Ramulus Cinnamomi cassiae, Scierotium Poriae cocos, Radix Albus paeoniae Lactiflorae, Cortex Radicis moutan, and Semen Pruni persicae | Gallic acid, paeonol, and paeoniflorin | Prepared by water distillation of Cortex Radicis moutan for volatile oil, followed by reflux extraction of the dregs with Ramulus Cinnamomi cassiae, Sclerotium Poriae cocos, Radix Albus paeoniae Lactiflorae, and Semen Pruni persicae in 90% ethanol twice and then water extraction twice, with all extracts combined and concentrated to 1.5 g/mL. | p-AKT (Ser473)↓, mTOR↓ | Apoptosis ↑ | [136] |
| Tou Nong San extract | Radix Astragaliseu Seu Hedysari 12 g, Angelica sinensis 9 g, Ligustici Chuanxiong 9 g, Spina Gleditsiae 4.5 g, and stir-baked Squama Manis 3 g | Calycosin-7-O-β-D-glucoside, formononetin-7-O-β-D-glucoside, senkyunolide I, calycosin, formononetin, astragaloside IV, Z-ligustilide | Obtained by twice refluxing the dried herbs in 80% ethanol (1:10, w/v) for 2 hours each, followed by solvent evaporation under reduced pressure, yielding 66.7 mg extract per gram of dried herbs. | p-PI3K↓, p-AKT↓, p-mTOR↓,p-p70S6K1↓ | Proliferation ↓, apoptosis ↑ | [137] |
| Galla chinensis ointment | Salvia miltiorrhiza, Clematis, black vinegar, Galla Chinensis, Cortex Lycii, and alum | No mentioned | Prepared by adding Galla chinensis medicinal solution to an emulsified base formed from oil and water phases at 80°C and stirring to room temperature. | p-AKT↓, p-mTOR↓, PTEN↑ | Proliferation ↓, apoptosis ↑ | [138] |
| Ziqi Ruangan decoction | -  (Only show abstract) | | | | | [139] |
| Yifei Sanjie formula | Astragalus membranaceus (Fisch.) Bunge 30g, Atractylodes macrocephala Koidz 20g, Saposhnikovia 10g, Fritillaria thunbergii 15g, Semen Sinapis 15g, Curcuma zedoaria rhizomes 15g and Panax notoginseng 9g | Ononin, Sissotrin, 3.alpha.-Mannobiose, Glycerophosphocholine , Ginsenoside Rg3, Ginsenoside Rb1, Formononetin, myo-Inositol, Stachyose, Salicylic acid, Glycitein, Ginsenoside Re, Galactonic acid, Thiamine, Adenine, Adenosine, Thymidine. Parthenolide. L-Asparagine. D-Arabinono-1,4-lactoned, D-Glucono-1,5-lactoned, Guanosine | Prepared by double decoction in a water bath, filtration, evaporation under reduced pressure below 55°C, freeze-drying, dissolution in distilled water, and sterilization through a 0.22 μm filter before intragastric administration. | p-PI3K↓, p-AKT↓, p-mTOR↓, LC3B-II↑, Beclin1↑ | Fibrosis ↓, inflammation ↓ | [140] |
| PSORI-CM02 | Rhizoma curcumae, Radix paeoniae rubra, Sarcandra glabra, Rhizoma smilacis glabrae, and Fructus mume | No mentioned | Extracted from pharmacopoeia-grade herbs using distilled water, followed by concentration and storage | p-PI3K↓, p-AKT↓, p-mTOR, Beclin-1↑ | Proliferation ↓, apoptosis ↑, autophagy ↑, inflammation ↓ | [141] |
| Jiang Zhi Granule | Herba gynostemmatis, Radix salviae, Rhizoma polygoni cuspidati, Herba artemisiae scopariae, and Folium nelumbinis | No mentioned | No mentioned | p-PI3K↓, p-mTOR↓, LC3-II/I↑, | Autophagy ↑, inflammation ↓ | [142] |
| Jinwu Jiangu Capsule | Cibotium barometz(L.)J. Sm., Homalomena occulta (Lour.) Schott., and Sabia parviflora wall. ex Roxb., Gardneria angustifolia Wall, Curcuma longa L., and Panaxnotoginseng (Burk.) F.H.Chen, Paeo- nia lactiflora Pall., Glycyrrhiza uralensis Fisch. | No mentioned | No mentioned | PI3K↓, AKT↓, mTOR↓, LC3-II↑ | Autophagy ↑, inflammation ↓ | [143] |
| Zi-Su-Zi decoction | Perillae Fructus 10g, Citri Exocarpium Rubrum 5g, Pinelliae Rhizoma 3g, Magnoliae Officinalis cortex 3g, Peucedani Radix 3g, Cinnamomi cortex 3g, Glycyrrhizae Radix et Rhizoma 3g | 52 main chemical components | Obtained by sequential decoction with strong and low fire, followed by a second extraction from the dregs, then concentrating and storing the combined liquid at 4°C, yielding 35% | p-PI3K↓, p-AKT1↓, p-mTOR↓ | Inflammation ↓, allergy ↓ | [144] |
| Xinfeng capsule | Coix lacryma-jobi var. ma-yuen, Astragalus membranaceus, Scolopendra subspinipes, Tripterygium wilfordii | No mentioned | Drug-containing serum obtained from rats orally administered Xinfeng capsule (0.2, 0.4, 0.8 mg/100 g) for 7 days, followed by blood collection, centrifugation, and serum inactivation at 56°C | PTEN↑, p-PI3K↓, p-AKT↓, p-mTOR↓ | Proliferation↓, inflammation↓ | [145] |
| Xuefu Zhuyu decoction | Prunus persica (L.) Batsch, Angelicae sinensis (Oliv.) Diels, Ligusticumi chuanxiong Hort., Carthamus tinctorius L., Paeonia lactiflora Pall., Rehmannia glutinosa Libosch., Citrus aurantium L., Bupleurum chinense DC., Platycodon grandiflorum (Jacq.) A. DC. | Hydroxysafflor yellow A and Amygdalin | No mentioned | p-AKT↓, p-mTOR↓, p-p70S6K↓ | Inflammation ↓ | [146] |
| Wuwei Wentong Chubi capsule | No mentioned | No mentioned | Prepared as capsules by the First Hospital Affiliated to Anhui University of Chinese Medicine | PI3K↓, p-AKT↓, p-mTOR↓, p-p70S6K↓, Beclin-1↑ | Autophagy↑, inflammation ↓ | [147] |
| Xuan-bai-cheng-qi decoction | Gypsum Fibrosum, Rheum officinale Baill, Prunus armeniaca L, Trichosanthes kirilowii Maxim | 43 compounds | No mentioned | p-PI3K↓, p-mTOR↓, HIF-1α↓, VEGF↓, PTEN↑, PI3K↓, AKT↓, mTOR↓ | Inflammation ↓ | [148] |
| Compound 511 | Panax ginseng C. Meyer (8.8%), Astragalus membranaceus (Fisch.) (18.2%), Datura metel Linn. (10.95%), Corydalis yanhusuo W. T. Wang (14.6%), Acanthopanar gracilistμlus W. W. Smith (10.95%), Ophiopogon japonicus (Linn. f.) Ker-Gawl. (10.95%), Gynostemma pentaphyllum (Thunb.) Makino (10.95%), Polygala arvensis Willd. (14.6%) | Quercetin, formononetin, kaempferol and β-sitosterol | Prepared by water decoction of medical herbs followed by concentration, vacuum drying, and storage at -20°C. | p-PI3K↓, p-AKT↓, p-mTOR↓ | Inflammation ↓, infection ↓, immune regulation | [149] |
| Effective-component compatibility of Bufei Yishen formula II | Ginseng Radix et Rhizoma 9g, Astragali Radix 15g, Corni Fructus 12g, Lycii Fructus 12g, Schisandrae Chinensis Fructus 9g, Fritillariae Thunbergii Bulbus 9g, Perillae Fructus 9g, Citri Reticulatae Pericarpium 9g, Epimedii Folium 9g, Paeoniae Rubra Radix 9g, Pheretima 12g, Ardisiae Japonicae Herba 15g | Icariin, astragaloside IV, schisandrin B, peimine, hesperidin, nobiletin, paeoniflorin and paeonol | No mentioned | EGFR↓, PI3K↓, AKT↓, mTOR↓ | Inflammation ↓, oxidation ↓ | [150] |
| Zhen-wu-tang | Aconitum carmichaelii Debeaux, Poria cocos (Schw.) Wolf, Atractylodes macrocephala Koidz, Paeonia lactiflora Pall and Zingiber officinale Roscoe in the ratio of 3:3:3:2:3 | Benzoylmesaconine, benzoylaconine, benzoylhypacoitine, gingerol-6, atractylenolide III, and atractylenolide II | Extracted by boiling the materials in water, followed by eight repeated 1.5-hour decoctions, and then concentrating the filtrate to 1.68 g/ml | p-PI3K↓, p-AKT↓, p-mTOR↓, p-AMPK↑ | Autophagy ↑, oxidation ↓ | [151] |
| The Jiedu Tongluo Baoshen formula | Rhizoma Dioscoreae Septemlobae, Radix Salviae Miltiorrhizae, Astragali Radix, Radix et Rhizoma Rhei, Semen Euryales, Corni Fructus, Ginseng Radix et Rhizoma, Folium Pyrrosiae and Hirudo in a weight ratio of 5: 5: 10: 2: 5: 4: 3: 4: 1 | Danshensu, verbascum glucoside, formononetin, panax notoginseng saponin R1, astragalus armour glycosides, rosemary acid | Extracted by boiling 117 g of crude drug mixture in 300 ml of distilled water at 100°C for 30 minutes twice | p-PI3K↓, p-AKT↓, p-mTOR↓, Beclin-1↑, LC3↑ | Autophagy ↑ | [152] |
| Dendrobium mixture | Dendrobium nobile Lindl. 15g, Astragalus membranaceus (Fisch.) Bunge 20g, Schisandra chinensis 8g, Radix Puerariae Lobatae 15g, Salvia miltiorrhiza 20g, Rehmanniae Radix 18g, and Rhizoma anemarrhenae 12g | No mentioned | Prepared by boiling the herbs in water for 1 hour after soaking, followed by filtration and concentration to 1.6 g/ml | p-PI3K↓, p-AKT↓, p-mTOR↓, LC3↑, Beclin-1↑ | Autophagy ↑ | [153] |
| Banxia-Houpu decoction | Pinellia ternata (Thunb.) Breit. (Araceae) 12g, Magnolia officinalis Rehd. et Wils. (Magnoliaceae) 9g, Poria cocos (Schw.) Wolf.(Polyporaceae) 12g, Zingiber officinale Rosc.(Zingiberaceae) 6g and Perilla frutescens (L.) Britt.(Lamiaceae) 15g | Magnolol, honokiol, succinic acid, rosmarinic acid and 6-gingerol | Prepared by immersing herbs in 10 volumes of water for 30 min, decocting at boiling for 40 min, then decocting residues with 8 volumes of water at simmer for 30 min, combining filtrates, centrifuging, concentrating to 100 mL, and storing at 4°C | p-PI3K↓, p-AKT↓, p-mTOR↓, Beclin-1↑, LC3-II/LC3-I↑ | Autophagy ↑, apoptosis ↓ | [154] |
| QiShenYiQi pill | Astragalus membranaceus (Fisch.) Bunge, Salvia miltiorrhiza Bunge, Panax pseudoginseng Wall. var. notoginseng (Burkill) Hoo et Tseng, Dalbergia odorifera | No mentioned | No mentioned | p-PI3K/PI3K↓, p-AKT/AKT↓, p-mTOR/mTOR↓, Beclin-1↑, LC3-II/LC3-I↑, PI3K↓, AKT↓, mTOR↓ | Autophagy ↑ | [155] |
| The Zuojin Pill | Coptis chinensis and Tetradium ruticarpum (A.Juss.) T.G.Hartley | Carbamazepine, Berberine hydrochloride, Coptis chinensis alkaloid, Palmatine, Magnolia alkaloid, Evodiamine, Rutaecarpine, Dehydroevodiamine | Prepared by soaking Coptis chinensis and Tetradium ruticarpum (6:1) in water, followed by double heat extraction and evaporation into dry powder. | PI3K↓, p-AKT↓, p-mTOR↓, P70S6K↓, PTEN↑, LC3-II↑, Beclin-1↑ | Inflammation ↓, autophagy ↑ | [156] |
| Shaoyao Decoction | Paeoniae Radix Alba, Angelicae Sinensis Radix, Coptidis Rhizoma, Scutellariae Radix, Glycyrrhizae Radix et Rhizoma, Arecae Semen, Aucklandiae Radix, Rhei Radix et Rhizome, Cinnanmomi Cortex in a ratio of 30:15:15:15:6:6:6:9:5 | Quercetin, Wogonin, Kaempferol, Baicalein, Aloe-emodin, Licochalcone a, Acacetin, Paeoniflorin, Naringenin, Isorhamnetin, Beta-sitosterol, Formononetin, Oroxylin a | Prepared by soaking the herb mixture in distilled water (1:10, w/v) for 30 min, boiling for 1 h, filtering, decocting the residue with distilled water (1:6, w/v), combining the filtrates, and concentrating to 1 g/mL at 60°C | p-PI3K ↓, p-AKT ↓, p-mTOR ↓, AKT1 ↓ | Inflammation ↓, apoptosis ↑ | [157] |
| JieZe-1 (derived from Yihuang Tang) | Phellodendri Chinensis cortex, Ginkgo Semen, and Solanum nigrum L., Taraxacum mongolicum Hand. - Mazz, Thlaspi arvense Linn., Dictamnus dasycarpus Turcz., Smilax glabra Roxb., Paeonia suffruticosa Andr., Mentha haplocalyx Briq. and Borneolum Syntheticum | Berberine, paeoniflorin, neoastilbin, astilbin, paeonol and dictamnine | Prepared by combining extracts of Phellodendri Chinensis Cortex, distillates of Moutan Cortex and Menthae Haplocalycis Herba, a concentrated decoction of other herbs, and borneol | p-PI3K↓, p-AKT↓, p-mTOR↓, LC3B-II↑ | Inflammation ↓, autophagy ↑ | [158] |
| Modified Xiaoyao San | Bupleurum chinense DC., Angelica sinensis (Oliv.) Diels, Atractylodes macrocephala Koidz., Paeonia lactiflora Pall., Poria cocos (Schw.) Wolf., Mentha haplocalyx Briq., Glycyrrhiza uralensis Fisch., Paeonia suffruticosa Andr., Gardenia jasminoides J.Ellis., Polygala tenuifolia Willd. and Acorus calamus var. Angustatus Besser. | Licochalcone, Quercetin, 6,8-dihydroxy-1,2,4-trimethoxyxanthone, Paeoniflorin, Licoricone, Galloylpaeoniflorin, 6-O-β-D-glucopyranosyllactinolide, Polygalaxanthone III, Ethyl gallate, Naringenin, Tryptophan, Mudanpioside I, Formononetin, Albiflorin, Oxypaeoniflora, Narcissoside, 16α,25-Dihydroxydehydroeburiconic acid, Kaempferol, 1-O-β-D-glucopyranosyl-8-O-benzoylpaeonisuffrone, Liquiritigenin, 3;7-Di-O-methylquercetin | Extracted by boiling water (1:8, twice), vacuum-dehydrated at 70 °C, and ground into powder with an extraction yield of 21.6%. | p-PI3K↓, p-AKT↓, p-mTOR↓ | Inflammation ↓, autophagy ↑ | [159] |
| Huang-Lian-Jie-Du-Decotion | Coptidis rhizoma, Scutellariae radix, Phellodendri cortex, and Gardeniae fructus in a weight ratio of 3:2:2:2 | Geniposide, Baicalin, Columbamine, Jatrorrhizine, Epiberberine, Coptisine, Palmatine, Berberine and Baicalein | Prepared by refluxing a 3:2:2:3 mixture of the four herbs with 70% ethanol and concentrating the decoction to give a 29.31% yield | p-mTOR ↓, p-JNK ↓, p-p38 ↓, p-ERK ↑, p-AKT ↑, Beclin-1 ↑, LC3-II/LC3-I ↑ | Inflammation ↓, autophagy ↑ | [160] |
| Qu-zhuo-tong-bi decoction | 60 g of Smilax glabra Roxb., 30 g of Dioscorea spongiosa, 15 g of Zea mays L., 30 g of Coix lacryma-jobi L., 18 g of Siegesbeckia orientalis L., 12 g of Curcuma longa L., 15 g of Taxillus chinensis, 18 g of Corydalis yanhusuo and 12 g of Fructus Citri Sarcodactylis | No mentioned | Extracted by pre-soaking in 10× deionized water for 1 h, decocting for 40 min, then boiling with 8× water for 30 min, followed by filtration, combination of filtrates, concentration, and storage at 4°C | p-PI3K↓, p-AKT↓, p-mTOR↓ | Inflammation ↓, regulates gut microbiota, and repairs intestinal barrier | [161] |
| Dingkun Pill | Panax ginseng C. A. Mey. 108g, Cervus Nippon Temminck 108g, Crocus sativus L. 16g, Panax notoginsen (Burk.) F.H.Chen 135g, Paeonia lactiflora Pall 162g, Rehmannia glutinosa Libosch 540g, Atractylodes macrocephala Koidz. 162g, Lycium barbarum L. 162g, Scutellaria baicalensis Georgi 11g, Cyperus rotundus L. 27g, Leonurus japonicus Houtt. 27g, Ligusticum chuanxiong Hort.16g, Asini Corii Colla 108g, Corydalis yanhusuo W.T.Wang 27g, Spatholobus suberectus Dunn 135g, Carthamus tinctorius L. 146g, Leonurus japonicus Houtt. 27g, Poria cocos (Schw.) Wolf 22g, Bupleurum chinense DC. 22g, Lindera aggregate (Sims) Kosterm. 16g, Eucommia ulmoides Oliv. 22g, Zingiber officinale Rosc. 22g, Amomum villosum Lour. 11g, Cyathula officinalis Kuan 16g, Cinnamomum cassia Presl 11g | No mentioned | No mentioned | Inhibit p-PI3K, p-AKT and p-mTOR | Apoptosis ↓ | [162] |
| Aurantii Fructus Immaturus + Atractylodis Macrocephalae Rhizoma | Aurantii Fructus Immaturus and Atractylodis Macrocephalae Rhizoma in a ratio of 2:1 | Narirutin, naringin, hesperidin, neohesperidin (from Aurantii Fructus Immaturus); Atractylenolide I, atractylenolide II, atractylenolide III (from Atractylodis Macrocephalae Rhizoma) | Authenticated, crushed into powder, soaked in water (1:10 w/v) for 30 min, decocted for 40 min, filtered, and the extract was freeze-dried into powder. The final concentration was 0.45 g/mL | - p-PI3K↓, p-AKT↓, p-mTOR↓, Beclin-1↓, LC3-II/LC3-I↓, Bcl-2↑ | Autophagy↓, apoptosis↓ | [163] |
| Astragali Radix + Salviae Miltiorrhizae Radix et Rhizoma | Astragali Radix + Salviae Miltiorrhizae Radix et Rhizoma | Calycosin-7-O-β-D-glucoside (from RA) and Sulfotanshinone sodium (from RS) | Air-dried, pulverized, reflux-extracted twice with water, concentrated, mixed with excipient (erythritol/stevioside, 99:1), and dried <60°C to form granules (2:1, herb:granule) | p-AMPKα1↑, sestrin-β↑, CaMKK-β↑, PI3K↓, AKT1↓, VEGFR2↓ | Anti-inflammatory ↓, cellular proliferation ↓, apoptosis ↑ | [164] |
| Glycyrrhiza uralensis Fisch + Zingiberis Rhizoma | Glycyrrhiza uralensis Fisch 12g and Zingiberis Rhizoma 6g, in a ratio of 2:1 | Liquiritin, glycyrrhizic acid, glycyrrhizin, 6-gingerol, and gingerone | Crushed, soaked in water (1:6 w/v) for 40 minutes, decocted twice (first for 30 minutes with vigorous fire, then for 30 minutes with mild fire), filtered, and the filtrate was concentrated to a final concentration of 1:3 (herb extract to water) | p-PI3K↓, p-AKT↓, p-mTOR↓ | Inflammatory↓, immune regulation↑ | [165] |
| Compound Kushen Injection | Sophora flavescens Aiton and Smilax glabra Roxb. | Matrine, oxymatrine, sophocarpine | Purchased from Shanxi Zhendong Pharmaceutical Co., Ltd., extracted by water or ethanol extraction and refined into injection | PI3K↓, p-AKT↓, AKT↓, mTOR↓, p-mTOR↓ | Proliferation↓, apoptosis↑, immunomodulation | [166] |
| Pingchuan Granules | Epimedium brevicornum Maxim., Fried Ephedra sinica Stapf, Astragalus membranaceus (Fisch.) Bunge, Pseudostellaria heterophylla (Miq.) Pax, Schisandra chinensis (Turcz.) Baill., Tussilago farfara L., Pheretima aspergillum (E. Perrier), Papaver somniferum L., Anemarrhena asphodeloides Bunge. | No mentioned | Obtained by orally administering Pingchuan Granules to Wistar rats twice daily for 3 days, followed by blood collection from the abdominal aorta 2 hours after the last dose. Serum was separated, inactivated at 56°C for 30 min, filtered through a 0.22 μm filter, and stored at -80°C | p-AKT↓, p-mTOR↓ | Autophagy↓, inflammatory↓, asthmatic↓ | [167] |
| Yiqi Jiedu Formula | Astragalus membranaceus (Fisch.) Bunge 30g, Codonopsis pilosula (Franch.) Nannf. 15g, Poria cocos (Schw.) Wolf 15g, Trichosanthes kirilowii Maxim. 10g, Coptis chinensis Franch. 10g, Hedyotis diffusa Willd. 10g, Glycyrrhiza uralensis Fisch. 6g | No mentioned | Prepared by decoction according to standard pharmaceutical methods, resulting in a solution containing 0.5 g crude drug per mL | p-PI3K↓, p-AKT↓, p-mTOR↓, Beclin-1↑, LC3B↑ | Proliferation↓, autophagy↑ | [168] |
| Shugan Jianpi Jiedu Formula | Bupleurum chinense DC. 10g, Curcuma aromatica Salisb. 10g, Poria cocos (Schw.) Wolf 10g, Atractylodes macrocephala Koidz. 10g, Scutellaria barbata D.Don 30g, Hedyotis diffusa Willd. 30g, Taraxacum mongolicum Hand.-Mazz. 15g, Prunella vulgaris L. 15g | No mentioned | Decocted twice with 10 times warm water, then the decoctions were combined, filtered, and concentrated to a solution containing 1 g crude drug per mL | ER mRNA↓, PI3K mRNA↓, AKT mRNA↓, mTOR mRNA↓ | Proliferation↓ | [169] |
| Shenghui Granules | Rehmannia glutinosa Libosch., Cornus officinalis Sieb. et Zucc., Polygala tenuifolia Willd., Ziziphus jujuba Mill. var. spinosa (Bunge) Hu ex H.F.Chow, Platycladus orientalis (L.) Franco, Poria cocos (Schw.) Wolf, Panax ginseng C.A.Mey., Acorus tatarinowii Schott, Sinapis alba L. | No mentioned | Dissolved in ultrapure water to a concentration of 0.702 g/mL for oral administration to mice. The original preparation process of the granules is not detailed | p-PI3K↓, p-AKT↓, p-mTOR↓, PI3K↓, AKT↓, mTOR↓, p62↓, Beclin-1↑, LC3 II/LC3 I↑ | Autophagy↑ | [170] |
| Peiyuan Kang’ai Decoction | Panax quinquefolius L. 15g, Curcuma aromatica Salisb. 12g, Astragalus membranaceus (Fisch.) Bunge 15g, Atractylodes macrocephala Koidz. 15g, Curcuma phaeocaulis Val. 15g, Hedyotis diffusa Willd. 15g, Poria cocos (Schw.) Wolf 15g, Magnolia officinalis Rehd. et Wils. 12g, Pinellia ternata (Thunb.) Breit. 12g, Crataegus pinnatifida Bunge 12g | No mentioned | Decocted and concentrated to a final concentration of 2 g/mL, stored at 4°C | p-PI3K↓, p-AKT↓, p-mTOR↓, p62↓, LC3 II/LC3 I↑ | Autophagy↑ | [171] |
| Modified Leweiyin Formula | Coix lacryma-jobi L. 30g, Dioscorea opposita Thunb. 30g, Atractylodes macrocephala Koidz. (stir-fried) 15g, Paeonia lactiflora Pall. (stir-fried) 15g, Curcuma aromatica Salisb. 15g, Isodon serra (Maxim.) Kudô 15g. | No mentioned | Decocted twice with water, filtered, and concentrated to a solution containing 1.26 g crude drug per mL | PI3K↓, AKT↓, mTOR↓ | Glycolysis↓ | [172] |
| Juantong Decoction | Caulis Spatholobi 20g, Rhizoma Smilacis Glabrae 20g, Radix Salviae Miltiorrhizae 15g, Radix Paeoniae Rubra 10g, Radix Angelicae Sinensis 10g, Notoginseng Powder 10g, Fructus Psoraleae 10g, Rhizoma Atractylodis Macrocephalae 10g, Herba Lycopi 10g, Resina Draconis 6g, Rhizoma Chuanxiong 6g, Radix Glycyrrhizae Praeparata 6g | No mentioned | Decocted with distilled water and concentrated to a final concentration of 4.29 g crude drug per mL | PI3K↓, AKT↓, mTOR↓, p70S6K mRNA↓ | Apoptosis↑ | [173] |
| Jiedu Huoxue Prescription | Flos Lonicerae Japonicae 10g, Radix Scrophulariae 10g, Radix Angelicae Sinensis 12g, Radix Salviae Miltiorrhizae 20g, Notoginseng Powder 6g, Radix Glycyrrhizae 6g | No mentioned | Decocted with water and concentrated to a final concentration of 2.7 g crude drug per mL | PI3K↓, AKT↓, mTOR↓, Beclin-1↑, LC3 II/LC3 I↑ | Autophagy↑, inflammation↓ | [174] |
| Zequ Mingshan Compound | Alisma orientale (Sam.) Juz. 15g, Monascus purpureus Went 6g, Cassia obtusifolia L. 15g, Crataegus pinnatifida Bge. 10g | No mentioned | Decocted twice with water (first with 8-fold volume for 1 hour, then with 6-fold volume for 1 hour), then the decoctions were combined and concentrated to solutions containing 1.5 g crude drug per mL (high dose) and 0.3 g crude drug per mL (low dose) | PI3K↓, AKT↓, mTOR↓ | Inflammation↓ | [175] |
| Qi Shen Di Huang granule | Astragalus membranaceus, Codonopsis pilosula, Atractylodes macrocephala, Rehmannia glutinosa, Cibotium barometz, Lycium barbarum, Morinda officinali, Polygonatum sibiricum, Dioscorea opposita, Glycyrrhiza uralensis, Angelica sinensis, Pheretima aspergillum, Uncaria rhynchophylla, Citrus reticulata, Bupleurum chinense, Cimicifuga foetida, Pueraria lobata, etc. | Quercetin, epigallocatechin-3-gallate (EGCG), luteolin, wogonin, kaempferol, fisetin | Prepared from a decoction of the herbal mixture, which is concentrated and dried into a powder extract. The extraction yield is 33.9%, meaning 1g of extract is equivalent to 2.95g of raw herbs | p-PI3K↓, p-AKT↓, p-mTOR↓, HIF1α↓ | Immunomodulation, glycolysis↓ | [176] |
| Xiaoqinglong Decotion | Ephedra sinica Stapf, Cinnamomum cassia Presl, Paeonia lactiflora Pall., Zingiber officinale Rosc., Pinellia ternata Breit., Schisandra chinensis Baill., Asarum heterotropoides Fr. Schmidt, Glycyrrhiza uralensis Fisch. (8 crude drugs, equal ratio except Schisandra half-dose) | Kaempferol, quercetin, baicalein, β-sitosterol, naringenin | Water-decocted 1 h, condensed to 0.16 g crude drug/mL; stored at 4 °C; used within 7 days | PI3K↓, AKT↓, mTOR↓ (protein & mRNA); TNF-α↓, IL-6↓, IL-8↓ | Inflammation↓ | [177] |
| Chang-An-Jun-Tai Decoction | Pseudostellaria heterophylla (Miq.) Pax, Poria cocos (Schw.) Wolf, Atractylodes macrocephala Koidz., Paeonia lactiflora Pall., Glycyrrhiza uralensis Fisch., Citrus reticulata Blanco (pericarp), Saposhnikovia divaricata (Turcz.) Schischk., Aucklandia lappa Decne., Coptis chinensis Franch | Berberine, kaempferol, naringenin, wogonin, atractylenolide II | Decocted with water, filtered, and concentrated into granules by Guangdong Yifang Pharmaceutical Co., Ltd. Specific details | p-PI3K↓, p-AKT↓, p-mTOR↓, PI3K mRNA↓, AKT mRNA↓, mTOR mRNA↓ | Antidiarrheal↑, inflammation↓ | [178] |
| Xiang Lian Pill | Coptis chinensis Franch. (Rhizoma Coptidis) 375 g, Aucklandia lappa Decne. (Radix Aucklandiae) 375 g, Myristica fragrans Houtt. (Semen Myristicae) 95 g, Nelumbo nucifera Gaertn. (Semen Nelumbinis) 185 g | Coptisine, palmatine chloride, berberine chloride, costunolide, dehydrocostus lactone, dehydrodiisoeugenol, and others (e.g., quercetin, luteolin) | Obtained by combined ethanol reflux and aqueous extraction of the crushed herbs, followed by freeze-drying | p-PI3K↓, p-AKT↓, p-mTOR↓, NF-κB p65↓, HIF-1α↓, LC3↑, Beclin-1↑ | Inflammation↓, autophagy↑ | [179] |

**Supplementary Method: Detailed Criteria for Evidence Strength Assessment**

| Dimension | High (H) | Medium (M) | Low (L) |
| --- | --- | --- | --- |
| Model Correlation | Studies using APDS-specific models:  • Primary immune cells from APDS patients.  • Genetically engineered APDS animal models.  • Models of primary immunodeficiency or lymphoma. | Studies using symptom-relevant models that recapitulate key APDS clinical features:  • Recurrent respiratory infections (e.g., pneumonia, sinusitis).  • Lymphoproliferation (splenomegaly, lymphadenopathy).  • Autoimmune diseases (e.g., autoimmune cytopenias).  • Enteropathy (e.g., diarrhea, colitis). | Studies using models with low pathological relevance to APDS:  • Standard cancer cell lines alone.  • Disease models not sharing features with APDS. |
| Mechanism Depth | Studies providing direct and comprehensive mechanistic insight:  • Target Engagement: Direct binding evidence (e.g., kinase assay, molecular docking, SPR).  • Pathway Mapping: Systematic analysis of upstream/downstream pathway components (e.g., PI3K, AKT, mTOR, p70S6K).  • Functional Validation: Genetic tools (e.g., knockdown/overexpression) confirming target necessity. | Studies demonstrating correlative pathway modulation:  • Systematic Western Blot analysis showing key phospho-protein changes.  • Gene expression analysis related to the pathway. | Studies with indirect or weak mechanistic evidence:  • Evidence only from semi-quantitative methods (e.g., immunohistochemistry).  • Predictions from network pharmacology without solid experimental validation. |
| Data Robustness | Studies demonstrating high reproducibility and rigor:  • Consistent effects across multiple cell or animal models.  • Clear dose-response and time-course relationships.  • Use of relevant positive controls (e.g., Rapamycin, Idelalisib).  • Sound statistics and adequate sample size. | Studies with moderate robustness:  • Dose-response/time-course shown in a single model.  • Inclusion of a positive control, though potentially suboptimal. | Studies with major methodological limitations:  • Conclusions based on a single concentration or time point.  • Lack of a positive control.  • Poor data presentation, making reproducibility uncertain. |
| Safety Profile | Studies providing substantial safety data:  • Preliminary in vivo pharmacokinetic data (e.g., bioavailability, half-life).  • Systematic in vitro and in vivo toxicity assessment, indicating a wide therapeutic window.  • Safety evaluation specific to the immune system. | Studies with basic safety indications:  • Clear in vitro cytotoxicity data on normal cells.  • In vivo studies reporting no significant changes in body weight or histopathology of major organs. | Studies with inadequate safety assessment:  • Safety not mentioned or not assessed.  • Efficacy only at high concentrations/doses with a narrow therapeutic window. |
| Standardization Level | Agents with well-defined composition:  • Single Compound: Chemically defined structure and high purity.  • Standardized Extract: Designated content of marker compounds.  • Formula: Use of standardized preparation and quality control. | Agents with partially characterized composition. | Agents with poorly defined composition:  • Use of complex, non-standardized crude extracts.  • Vague or unclear preparation methods. |

**Rules for Deriving the Overall Evidence Intensity:**

The Overall Intensity is an integrative judgment based on the scores across all five dimensions, with primary weight given to the three core dimensions: Model Correlation, Mechanism Depth, and Safety Profile.

• High (H) Overall Intensity:

Prerequisite: Safety Profile is not Low (L).

Core Requirement: At least one of the core dimensions (Model Correlation or Mechanism Depth) is rated High (H), and the other is not Low (L).

Supporting Context: The other dimensions (Data Robustness, Standardization) are mostly rated High (H) or Medium (M).

• Medium (M) Overall Intensity:

Situation A (Well-Balanced): All dimensions are rated as Medium (M).

Situation B (Strengths with Weaknesses): One core dimension is High (H) but the other is Low (L).

• Low (L) Overall Intensity:

Situation A (Core Deficit): Both Model Correlation and Mechanism Depth are rated Low (L).

Situation B (Critical Safety Concern): Safety Profile is rated Low (L).

Situation C (Generally Weak): The majority of dimensions, especially the core ones, are rated Low (L).

**Supplementary Table 12. Star Candidate Evaluation Form**

| Category | Name | Model correlation | Mechanism depth | Data Robustness (Multiple models/ Clear dose gradient effect and time effect/ Positive controls/ Adequate sample size) | Safety (Toxicity assessment/ Pharmacokinetic data) | Standardization level | Overall intensity |
| --- | --- | --- | --- | --- | --- | --- | --- |
| Herbs | Sanguisorba officinalis L. | L (②/A549, H1299) | M (Western Blot) | H (Y/Y/Y/Y) | M (Y/N) | M | M |
|  | Radix Astragali fermented with Paecilomyces cicadae | L (③) | H (Western Blot, LY294002/3-MA) | H (Y/Y/Y/Y) | M (Y/N) | H | M |
|  | Aconitum pendulum Busch | M (①/RA: arthritis, autoimmune response ) | M (Molecular docking, In vivo testing) | M (N/Y/Y/Y) | M (Y/N) | M | M |
|  | Orthosiphon stamineus Benth. | L (③) | M (Western Blot, RT-PCR) | M (Y/Y/N/Y) | L (N/N) | L | L |
|  | Ainsliaea fragrans Champ. | L (③) | M (Western Blot, RT-PCR) | M (N/Y/Y/Y) | M (Y/N) | M | M |
| Flavonoids | Myricetin | L (②/AGS) | H (Western Blot, LY294002/3-MA) | H (Y/Y/Y/Y) | M(Y/N) | H | M |
|  | Baicalein | L (②/MCF, MDA-MB-231) | H (Western Blot, RT-PCR, LY294002) | H (Y/Y/Y/Y) | M(Y/N) | H | M |
|  | Quercetin | L (②/A549, H1299) | H | M (Y/Y/N/Y) | M(Y/N) | H | M |
|  | Kaempferol | L (②/KB cell) | H | M (N/Y/Y/Y) | L (N/N) | H | L |
|  | Isorhamnetin | L (②/HT-29, HCT116, SW480) | H (Western Blot, LY294002, Rapamycin) | H (Y/Y/Y/Y) | M(Y/N) | H | M |
|  | Fisetin | L (②/PANC-1, Patu-8988) | M (Western Blot) | M (Y/Y/N/Y) | M(Y/N) | H | M |
|  | Apigenin | L (②/HepG2) | H (Western Blot, LY294002) | H (Y/Y/Y/Y) | M(Y/N) | H | M |
|  | Nobiletin | L (②/PDX) | H | M (Y/Y/Y/N) | M(Y/N) | H | M |
|  | Lupiwighteone | L (②/MCF, MDA-MB-231) | H (Western Blot, LY294002) | M (Y/Y/N/Y) | M(Y/N) | H | M |
|  | Delphinidin | L (②/SKOV3) | H (Western Blot, LY294002) | H (Y/Y/Y/Y) | L (N/N) | H | L |
|  | Pelargonidin | M (①/Glioma model) | M (Western Blot) | M (N/Y/Y/Y) | L (N/N) | H | L |
|  | Genistein | L (②/MCF, MDA-MB-231) | M (Western Blot) | M (Y/Y/N/Y) | M(Y/N) | H | M |
|  | Brusatol | L (②/Hep3B, Huh7, LM3, Bel7404) | M (Western Blot) | M (Y/Y/N/Y) | M(Y/N) | H | M |
|  | Hesperidin | L (②/Rat liver cancer model) | M (Western Blot) | M (Y/N/N/Y) | L (N/N) | H | L |
|  | Genkwanin | L (②/MDA-MB-231, MCF-7, A549) | H | M (Y/Y/N/Y) | M(Y/N) | H | M |
|  | Pectolinarigenin | L (②/AGS, MKN28) | M (Western Blot, RT-PCR) | M (Y/Y/N/Y) | L (N/N) | H | L |
|  | Silibinin | L (②/MCF-7) | M (Western Blot, RT-PCR) | M (N/Y/N/Y) | L (N/N) | H | L |
|  | Synthesized silymarin-mediated | L (②/AGS, HepG2, A549, Hela) | H (Western Blot, 740Y-P, CQ) | M (Y/Y/Y/N) | M(Y/N) | H | M |
|  | Isoorientin | L (②/HepG2) | H | M (N/Y/N/Y) | L (N/N) | H | M |
|  | Total flavonoid aglycones extract in Radix Scutellariae | L (②/BxPC3, PANC1) | H (Western Blot, SH-6, 3-MA) | M (Y/Y/Y/N) | M(Y/N) | H | M |
|  | Luteoloside | L (②/A549, H292) | H (Western Blot, 3-MA, Rapamycin) | H (Y/Y/Y/Y) | M(Y/N) | H | M |
|  | Quercetin-3-methyl ether | L (②/KYSE450, KYSE510) | H | H (Y/Y/Y/Y) | M(Y/N) | H | M |
|  | Farrerol | L (③) | M (Western Blot) | M (N/Y/N/Y) | L (N/N) | H | L |
|  | Ampelopsin | M (①/Inflammatory model) | H (Western Blot, LY294002) | M (N/Y/Y/Y) | M(Y/N) | H | H |
|  | Oroxin B | L (③) | M (Western Blot) | H (Y/Y/Y/Y) | M(Y/N)  ≤160μM non-toxic | H | M |
|  | Oroxylin A | M (①/Inflammatory model) | M (Western Blot) | M (N/Y/N/Y) | L (N/N) | H | L |
|  | Pogostone | L (②/HCT116) | M (Western Blot) | M (N/Y/Y/N) | M (Y/N) | H | M |
|  | Calycosin | L (③) | M (Western Blot) | M (Y/Y/N/Y) | M(Y/N) | H | M |
|  | Cyanidin-3-glucoside | L (②/H1299, A549) | M (Western Blot, RT-qPCR) | M (Y/Y/N/Y) | L(Y/N) 80 μM exhibits mild toxicity to normal lung epithelial cells BEAS-2B. | H | L |
|  | Puerarin | L (②/H1299, A549) | M (Western Blot, RT-qPCR) | H (Y/Y/Y/Y) | M(Y/N) | H | M |
|  | Dihydromyricetin | M (①/Diarrhoea, Inflammatory model) | H (Western Blot, 3-MA, 704Y-P) | H (Y/Y/Y/Y) | M(Y/N) | H | H |
| Terpenoids | Bruceine D | L (②/MDA-MB-231) | M (Western Blot) | M (N/Y/N/Y) | L (N/N) | H | L |
|  | Betulinic acid | L (②/MCF-7, MDA-MB-231) | H (Western Blot, LY294002) | H (Y/Y/Y/Y) | M(Y/N) | H | M |
|  | Stigmasterol | L (②/HUVEC) | M (Western Blot, RT-PCR,ELISA ) | M (Y/N/N/Y) | M(Y/N) | H | M |
|  | Jolkinolide B | L (②/TU212, TU177, Hep-2) | H (Western Blot, LY294002) | H (Y/Y/Y/Y) | L (N/N) | H | L |
|  | Afrocyclamin A | L (②/DU145, LNCaP, PC-3) | H (Western Blot, functional verification) | H (Y/Y/Y/Y) | M(Y/N) | H | M |
|  | Celastrol | M (①/Inflammatory model) | H (Western Blot, 3-MA) | M (N/Y/Y/Y) | M(Y/N) | H | H |
|  | Ganoderma tsugae | L (②/K562, U937, HL-60) | H (Western Blot, RT-PCR, 3-MA) | M (Y/Y/N/Y) | M(Y/N) | L | M |
|  | Ursolic acid | L (②/SKOV-3) | M (Western Blot) | M (N/Y/Y/Y) | M(Y/N) | H | M |
|  | Demethylzeylasteral | L (②/MKN-45) | M (Western Blot) | M (N/Y/N/Y) | L(N/N) | H | L |
|  | Germacrone | L (③) | M (Western Blot) | M (Y/Y/N/Y) | L (N/N) | H | L |
|  | Helenalin | L (③) | M (Western Blot) | M (N/Y/Y/Y) | M(Y/N) | H | M |
|  | Andrographolide | M (①) | H (Western Blot, 3-MA) | H (Y/Y/Y/Y) | M(Y/N) | H | H |
|  | Beta-Elemene | L (②/A549) | M (Western Blot) | H (Y/Y/Y/Y) | M(Y/N) | H | M |
|  | An ethyl acetate fraction of Artemisia capillaris (ACE-63) | L (②/HCC) | M (Western Blot) | M (Y/Y/N/Y) | L (N/N) | M | L |
|  | Safranal | L (②/GBM#P3, BG7) | H (Western Blot, SC79) | H (Y/Y/Y/Y) | L (N/N) | H | L |
|  | TEEG (3β,16β,23-trihydroxy-13,28-epoxyurs-11-ene-3-O-β-D-glucopyranoside) | L (②/A549) | M (Western Blot) | M (N/Y/N/Y) | L(N/N) | H | L |
|  | Gypenosides | L (②/T24, 5637) | M (Western Blot, RT-qPCR) | M (Y/Y/N/Y) | M(Y/N) | M | M |
|  | Ginsenoside Rg3 | L (②/AGS) | M (Western Blot, RT-qPCR) | M (Y/Y/N/Y) | L(Y/N) High concentrations (100–200 μg/ml) are toxic, while low concentrations (≤50 μg/ml) are not significantly toxic | H | L |
|  | Astragaloside IV | L (③) | M (Western Blot) | M (Y/N/Y/Y) | M(Y/N) | H | M |
|  | Astragaloside II | L (②/786-O) | M (Western Blot) | M (Y/Y/N/Y) | L(N/N) | H | L |
|  | Panax Notoginseng Saponins | L (③) | M (Western Blot) | M (N/N/Y/Y) | L(N/N) | L | L |
| Steroids | Rhizoma Paridis saponins | L (②) | M (Western Blott, RT-PCR) | M (N/N/Y/Y) | M(Y/N) | M | M |
|  | The saponin DT-13 | L (②/MDA-MB-231, MDA-MB-468) | H (Western Blot, RT-qPCR, LY294002) | M (Y/Y/N/Y) | M(Y/N) | H | M |
|  | Bufalin | L (②/ACHN) | M (Western Blott) | M (N/Y/N/Y) | M(Y/N) | H | M |
|  | Polyphyllin VI | L (②/ECA-109) | M (Western Blott) | M (N/Y/N/Y) | L(N/N) | H | L |
| Alkaloids | Krukovine | L (②/AsPC-1, Panc-1, MiaPaCa-2) | M (Western Blot, RT-qPCR) | M (Y/Y/N/Y) | L(N/N) | H | L |
|  | Tetrandrine | L (②/SW620) | M (Western Blot, RT-qPCR) | M (Y/Y/N/Y) | M(Y/N) | H | M |
|  | Matrine | L (②/SK-N-AS, SK-N-DZ) | M (Western Blot) | M (Y/Y/N/Y) | L(N/N) | H | L |
|  | Oxymatrine | L (②/U251MG) | M (Western Blot) | M (N/Y/N/Y) | L(N/N) | H | L |
|  | Berberine | M (①/Inflammatory model) | M (Western Blot, RT-qPCR) | M (Y/Y/N/Y) | L(N/N) | H | L |
|  | Aconiti Lateralis Radix Praeparata alkaloids | L (②/A549) | M (Western Blot) | H (Y/Y/Y/Y) | M (Y/N) | H | M |
|  | Toddalia asiatica alcohol extract | M (①/Inflammatory model) | H (Western Blot, 740Y-P/3-MA) | M (N/Y/N/N) | M (Y/N) | M | H |
|  | Brucine | L (②/U251) | H (Western Blot, PI3K inhibitors) | M (N/Y/N/N) | M (Y/N) | H | M |
| Phenols | Deoxypodophyllotoxin | L (②/U2OS) | M (Western Blot) | M (Y/Y/N/Y) | L(N/N) | H | L |
|  | Rosemary extract | L (②/A549) | M (Western Blot) | M (N/Y/Y/Y) | L(N/N) | H | L |
|  | Quercetin-3-O-glucoside | L (②/CFPAC-1, SNU-213, Panc-1) | M (Western Blot) | M (N/Y/Y/Y) | L(N/N) | H | L |
|  | Licochalcone A | L (②/MCF-7) | M (Western Blot) | M (N/Y/N/Y) | L(N/N) | H | L |
|  | Curcumin | L (③) | M (Western Blot) | H (Y/Y/Y/Y) | M(Y/N) | H | M |
|  | Pterostilbene | L (②/GBC-SD, SGC-996, NOZ) | M (Western Blot) | M (Y/Y/N/Y) | M(Y/N) | H | M |
|  | Epigallocatechin-3-gallate (EGCG) | M (①//Inflammatory model) | H | M (Y/Y/N/Y) | M(Y/N) | H | H |
|  | Osthole (7-methoxy-8-isopentenoxycoumarin, C15H16O3) | L (②/Y-79) | M (Western Blot, RT-qPCR) | M (Y/Y/N/Y) | L(N/N) | H | L |
|  | Resveratrol (3,4,5-trihydroxystilbene) | L (②/T24, 5637, EJ) | M | M (Y/Y/N/Y) | M(Y/N) | H | M |
|  | 1,2,3,4,6-penta-O-galloyl-beta-D-glucose (PGG) | L (②/HUVEC) | M (Western Blot) | M (N/Y/Y/Y) | L(N/N) | H | L |
|  | Ellagic acid (EA, 4,4′,5,5′,6,6′-hexahydroxydiphenic acid 2,6,2′,6′-dilactone) | L (②/BHT-101, Nthy-ori3-1) | M (Western Blot) | M (N/Y/N/Y) | M(Y/N) | H | M |
|  | Salidroside | L (②/AGS) | M (Western Blot) | M (N/N/N/Y) | L(N/N) | H | L |
| Quinones | Salvianolic acid A(Sal A) | L (②/U87) | M (Western Blot) | M (N/Y/N/Y) | M(Y/N) | H | M |
|  | Salvianolic acid B (Sal B) | L (②/A549) | M (Western Blot) | M (N/Y/Y/Y) | L(N/N) | M | L |
|  | Mollugin | L (②/HepG2, MCF-7, A549) | H (Western Blot, LY294002) | H (Y/Y/Y/Y) | M(Y/N) | H | M |
|  | Shikonin | L (②/MCF-7) | M (Western Blot) | M (N/Y/N/Y) | L(N/N) | H | L |
|  | Coenzyme Q0 | L (②/U87MG, GBM8401) | H (Western Blot, Z-VAD-FMK, 3-MA, CQ,NAC ) | H (Y/Y/Y/Y) | M(Y/N) | H | M |
|  | Tanshinone IIA | L (②/H1688, H446) | H (Western Blot, LY294002) | H (Y/Y/Y/Y) | M(Y/N) | H | M |
|  | Cryptotanshinone | L (②/CT26) | M (Western Blot) | M (N/Y/N/Y) | M(Y/N) | H | M |
|  | Danshen injection | L (②/MPC-5) | H (Western Blot, rapamycin) | H (Y/Y/Y/Y) | M(Y/N) | H | M |
|  | Hydroxysafflor yellow A | L (③) | H (Western Blot, LY294002) | H (Y/Y/Y/Y) | M(Y/N) | H | M |
|  | Emodin | L (③) | M (Western Blot) | M (Y/Y/N/Y) | L(N/N) | H | L |
|  | Rhein | M (①/Inflammatory model) | M (Western Blot) | H (Y/Y/Y/Y) | M(Y/N) | H | H |
| Lignans | Deoxypodophyllotoxin (anthricin) | L (②/U2OS) | M (Western Blot) | M (N/Y/N/Y) | M(Y/N) | H | M |
|  | Honokiol | L (②/MG-63) | M (Western Blot) | M (N/Y/N/Y) | L(N/N) | H | L |
|  | Schisandrin B | L (②/143B, MG63, Saos2, U2OS) | H (Western Blot, BML284, SC79) | M (Y/Y/N/Y) | M(Y/N) | H | M |
| Other classes of drugs or unknown | Spatholobus suberectus Column Extract | L (②/MCF-7) | M (Western Blot) | M (N/Y/N/Y) | L(N/N) | M | L |
|  | Celastrus orbiculatus ethyl acetate extracts | L (②/HT-29) | H (Western Blot, 3-MA, rapamycin) | M (N/Y/N/Y) | L(N/N) | H | L |
|  | Centipeda minima ethanol extracts | L (②/143B, MG63, Saos2, U2OS) | H (Western Blot, BML284, SC79) | M (Y/Y/N/Y) | M(Y/N) | H | M |
|  | Zanthoxylum bungeanum seed oil | L (②/Hep-2) | M (Western Blot, qRT-PCR) | M (N/Y/Y/Y) | L(N/N) | M | L |
|  | Sarcandra Glaba | L (②/DU-145) | M (RT-PCR) | M (N/Y/Y/N) | L (N/N) | L | L |
|  | Marsdenia tenacissima extract | L (②/H460, H1975) | M (Western Blot) | M (Y/Y/N/Y) | M(Y/N) | M | M |
|  | Kochia scoparia seed extract | L (②/LNCaP, PC-3) | M (Western Blot, ELISA) | M (Y/Y/N/Y) | M(Y/N) | M | M |
|  | Forsythia suspensa extract | L (②/SW480) | M (Western Blot) | M (N/Y/N/Y) | L(N/N) | M | L |
|  | Prunella vulgaris extract | M (①/LY8, RAJI, DOHH2) | M (Western Blot) | H (Y/Y/Y/Y) | M(Y/N) | M | H |
| Fomula | The Qingyihuaji decoction | L (②/PANC-1, MIA PaCa-2) | M (Western Blot, qRT-PCR) | M (Y/Y/N/Y) | M(Y/N) | H | M |
|  | Sijunzi Decoction | L (②/HCT116, LOVO) | H (Western Blot, LY294002, IGF-1) | H (Y/Y/Y/Y) | M(Y/N) | M | M |
|  | Wei-Tong-Xin | L (②/HCT116) | H (Western Blot, LY294002, RT-qPCR) | M (N/Y/Y/Y) | L(N/N) | M | L |
|  | Fuzheng Jiedu Decoction | L (②/SKOV3, 3AO) | M (Western Blot, LY294002, IGF-1) | H (Y/Y/Y/Y) | M(Y/N) | M | M |
|  | modified Jianpi Yangzheng decoction | L (②/MGC-803, SGC-7901, BGC-823) | M (Western Blot) | H (Y/Y/Y/Y) | M(Y/N) | M | M |
|  | Weipixiao decoction | L (②/GPL) | M (Western Blot) | M (N/Y/Y/Y) | L(N/N) | M | L |
|  | Kanglaite® injection | L (②/PANC-1) | M (Western Blot, RT-PCR) | M (N/Y/N/Y) | L(N/N) | H | L |
|  | Gubenyiliu formula II | L (②/MDA-MB-231, MCF-7, 4T1) | M (Western Blot) | M (N/Y/Y/Y) | L(N/N) | H | L |
|  | NPC01 | L (②/5-8F, NP69) | H (Western Blot, LY294002, Rapamycin) | M (N/Y/N/Y) | L(N/N) | M | L |
|  | CFF-1 | L (②/LNCaP, PC3, DU145, CWR22Rv1) | M (Western Blot) | M (Y/Y/N/Y) | M(Y/N) | L | L |
|  | NSENL | L (②/A549, DDP) | M (Western Blot, RT-PCR) | M (N/Y/N/Y) | L(N/N) | M | L |
|  | Xihuang Pill | L (②/SMMC-7721) | M (Western Blot, RT-qPCR) | M (N/Y/N/Y) | M(Y/N) | M | M |
|  | Guizhi Fuling Wan | L (②/SKOV3, DDP) | M (Western Blot) | M (N/Y/Y/Y) | M(Y/N) | H | M |
|  | Tou Nong San extract | L (②/LoVo) | M (Western Blot) | M (Y/Y/N/Y) | M(Y/N) | M | M |
|  | Galla chinensis ointment | L (③) | M (Western Blot, RT-qPCR) | M (Y/Y/N/Y) | M(Y/N) | M | M |
|  | Ziqi Ruangan decoction | No full text | | | | | |
|  | Yifei Sanjie formula | L (③) | M (Western Blot, RT-qPCR) | H (Y/Y/Y/Y) | M(Y/N) | M | M |
|  | PSORI-CM02 | L (②/HepG2) | H (Western Blot, Rapamycin) | H (Y/Y/Y/Y) | M(Y/N) | M | M |
|  | Jiang Zhi Granule | L (③) | M (Western Blot, RT-PCR) | M (Y/Y/N/Y) | M(Y/N) | M | M |
|  | Jinwu Jiangu Capsule | M (①/Inflammatory model) | M (Western Blot, RT-qPCR) | M (N/Y/Y/Y) | L(N/N) | L | L |
|  | Zi-Su-Zi decoction | M (②/CVA) | M (Western Blot, RT-qPCR) | M (N/Y/Y/Y) | M(Y/N) | M | H |
|  | Xinfeng capsule | L (③) | M (Western Blot, RT-PCR) | M (N/Y/N/Y) | M(Y/N) | L | M |
|  | Xuefu Zhuyu decoction | L (③) | M (Western Blot) | M (N/Y/N/Y) | L(N/N) | M | L |
|  | Wuwei Wentong Chubi capsule | M (①/RA) | M (Western Blot, RT-qPCR) | M (N/N/Y/Y) | L(N/N) | L | L |
|  | Xuan-bai-cheng-qi decoction | M (①/Acute lung injury) | M | H (Y/Y/Y/Y) | L(N/N) | M | L |
|  | Compound 511 | M (①/Pneumonia model) | M (Western Blot, RT-qPCR) | H (Y/Y/Y/Y) | M(Y/N) | H | H |
|  | Effective-component compatibility of Bufei Yishen formula II | M (①/COPD) | M (Western Blot, RT-qPCR) | M (N/Y/Y/Y) | M(Y/N) | M | M |
|  | Zhen-wu-tang | M (①/CGN) | M (Western Blot, RT-qPCR) | M (Y/Y/N/Y) | L(N/N) | M | L |
|  | The Jiedu Tongluo Baoshen formula | L (③) | M (Western Blot, RT-qPCR) | M (N/Y/Y/Y) | L(N/N) | M | L |
|  | Dendrobium mixture | L (③) | M (Western Blot, RT-qPCR) | M (N/Y/Y/Y) | L(N/N) | M | L |
|  | Banxia-Houpu decoction | L (③) | M (Western Blot) | M (N/Y/N/Y) | L(N/N) | M | L |
|  | QiShenYiQi pill | L (③) | M (Western Blot) | M (N/Y/Y/Y) | L(N/N) | M | L |
|  | The Zuojin Pill | L (③) | M (Western Blot, RT-qPCR) | M (N/Y/Y/Y) | L(N/N) | M | L |
|  | Shaoyao Decoction | M (①/UC) | M (Western Blot) | M (N/Y/N/Y) | M(Y/N) | H | H |
|  | JieZe-1 (derived from Yihuang Tang) | L (③) | M (Western Blot, RT-qPCR) | M (N/Y/Y/Y) | M(Y/N) | M | M |
|  | Xiaoyao San | L (③) | H (Western Blot, 3-MA) | M (N/Y/Y/Y) | M(Y/N) | M | M |
|  | Huang-Lian-Jie-Du-Decotion | L (③) | M (Western Blot) | M (N/Y/N/Y) | L(N/N) | M | L |
|  | Qu-zhuo-tong-bi decoction | L (③) | H | M (N/Y/Y/Y) | M(Y/N) | M | M |
|  | Dingkun Pill | L (③) | M (Western Blot, RT-qPCR) | M (N/Y/Y/Y) | L(N/N) | L | L |
|  | Aurantii Fructus Immaturus + Atractylodis Macrocephalae Rhizoma | L (③/ICCs) | M (Western Blot) | M (N/Y/N/N) | L (N/N) | M | L |
|  | Astragali Radix + Salviae Miltiorrhizae Radix et Rhizoma | L (③) | M (Western Blot) | H (Y/Y/Y/Y) | M (Y/N) | M | M |
|  | Licorice + Rhizoma Zingiberis | M (①/Airway inflammation) | M (Western Blot) | M (Y/Y/N/Y) | M(Y/N) | M | M |
|  | Compound Kushen Injection | M (①/EL4) | M (Western Blot) | H (Y/Y/Y/Y) | M(Y/N) | M | H |
|  | Pingchuan Granules | M (①/16HBE) | H (Western Blot, LY294002) | M (N/Y/Y/Y) | M(Y/N) | M | H |
|  | Yiqi Jiedu Formula | L (②/CNE-2Z) | H (Western Blot, 3-MA) | M (N/Y/Y/Y) | L(N/N) | L | L |
|  | Shugan Jianpi Jiedu Formula | L (②) | L (RT-PCR) | M (N/Y/Y/Y) | L(N/N) | L | L |
|  | Shenghui Granules | L (③) | M (Western Blot) | M (Y/N/Y/Y) | L(N/N) | L | L |
|  | Peiyuan Kang’ai Decoction | L (②) | M (Western Blot) | M (N/N/Y/Y) | L(N/N) | L | L |
|  | Modified Leweiyin Formula | L (③/CAG) | M (Western Blot, qRT-PCR) | M (N/N/Y/Y) | L(N/N) | L | L |
|  | Juantong Decoction | L (③/EMS) | M (LY294002, Real-time PCR) | M (N/N/Y/Y) | L(N/N) | L | L |
|  | Jiedu Huoxue Prescription | L (③/AS) | H (Western Blot, Rapamycin) | M (N/N/Y/Y) | L(N/N) | L | L |
|  | Zequ Mingshan Compound | L (③/NASH) | M (Western Blot, qRT-PCR) | M (N/N/Y/Y) | L(N/N) | L | L |
|  | Qi Shen Di Huang granule | M (①/EAMG) | M (Western Blot, qRT-PCR) | M (N/Y/Y/Y) | M(Y/N) | M | M |
|  | Xiaoqinglong Decotion | M (①/RRTI) | M (Western Blot, qRT-PCR) | M (N/Y/N/Y) | M(Y/N) | L | M |
|  | Chang-An-Jun-Tai Decoction | M (①/IBS-D) | H (Western Blot, qRT-PCR, LY294002) | M (N/Y/Y/Y) | M(Y/N) | M | H |
|  | Xiang Lian Pill | M (①/UC) | M (Western Blot, qRT-PCR) | H (Y/Y/Y/Y) | M(Y/N) | M | H |

H: High, M: Medium, L: Low; N: no; Y: yes; ①: Pathophysiologically relevant models ②: Standard cancer cell lines ③: Pathophysiologically divergent models
